# Unveiling the Roles of Pt and CeO_2_ during Solvent-Free Amide Hydrogenation Utilizing *Operando* Photoelectron Photoionization Coincidence Spectroscopy

**DOI:** 10.1021/acscatal.4c07955

**Published:** 2025-04-02

**Authors:** Xinbang Wu, Rosie J. Somerville, Andras Bodi, Roland C. Turnell-Ritson, Zihao Zhang, Jan Romano-deGea, Jaques-Christopher Schmidt, Patrick Hemberger, Paul J. Dyson

**Affiliations:** † Institute of Chemical Sciences and Engineering, École Polytechnique Fédérale de Lausanne(EPFL), Lausanne 1015, Switzerland; ‡ Laboratory of Synchrotron Radiation and Femtochemistry, Paul Scherrer Institute, Villigen 5232, Switzerland

**Keywords:** heterogeneous catalysis, *operando* studies, solvent-free, amide hydrogenation, amide hydrogenolysis, bifunctional catalysis

## Abstract

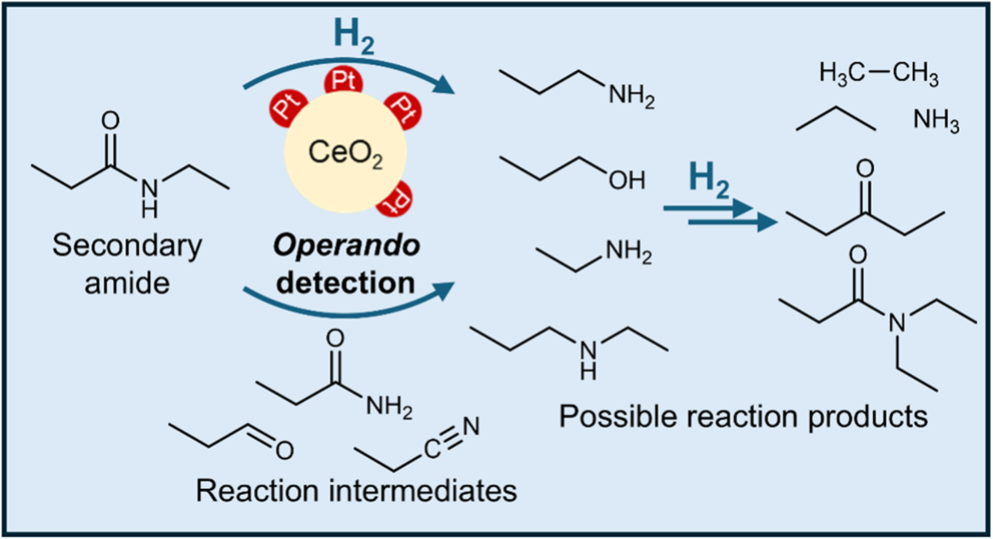

The hydrogenation of amides to amines is an important reaction in the synthesis of building blocks in organic chemistry. However, the solvent-free hydrogenation of amides (requiring heterogeneous catalysts and high reaction temperatures) is challenging, with low selectivity obtained due to further hydrogenolysis of the alcohol and amine products. Such behavior was observed for a CeO_2_-supported Pt catalyst (Pt/CeO_2_). Although it is known that the support promotes the adsorption of the amide on the catalyst, and Pt facilitates hydrogenation, their individual roles in amide conversion and how they impact product selectivity remain unclear. Using *operando* photoelectron photoionization coincidence (PEPICO) spectroscopy, the conversion of *N*-ethylpropionamide was studied over both Pt/CeO_2_ and CeO_2_. The experiments reveal that Pt is active for amide hydrogenation below 150 °C to produce amines with high selectivity. The Lewis acidic support (CeO_2_) is also active at higher temperatures, producing ethylamine and propanal, while being vulnerable to coking. Although Pt nanoparticles mitigate coke formation, they also catalyze the hydrogenolysis of the amine products above 150 °C. This study provides insights on bifunctional metal oxide--metal nanoparticle catalysts in amide hydrogenation to facilitate the design of superior catalysts.

## 1 Introduction

The hydrogenation of amides is important in fields ranging from pharmaceutical synthesis to plastic recycling.^1^ Amines are essential building blocks in organic chemistry, and the use of green hydrogen as hydrogen source promises a sustainable avenue to their production from amides.^2^ Both homogeneous and heterogeneous catalysts have been developed for the hydrogenation of acyclic amides,^3−5^ cyclic or aryl amides,^4−7^ and, more recently, for the recycling of polyamides.^8−11^ A metal site is typically employed for the adsorption and transfer of hydrogen, where the active species and the coordination environment play a crucial role in determining the selectivity for C–O or C–N bond hydrogenation.^12−14^ (Herein we use *hydrogenation* to refer to the cleavage of a C–O or C–N bond to form amines or alcohols, and *hydrogenolysis* to refer to the cleavage of a C–heteroatom bond resulting in a loss of amine or alcohol functional group). Recently, there has been a strong incentive to hydrogenate polyamides, which can be sourced from abundant waste biomass (e.g., keratin) or plastic (e.g., nylon), as a pathway to platform chemicals such as amines. However, polyamides contain repeating units of secondary amides, which are particularly challenging to hydrogenate with high selectivity compared to primary or tertiary amides.^15^ Thus, developing new catalysts capable of achieving high conversion and selectivity in the hydrogenation of secondary amides is important for sustainable processes.

**Scheme 1:**
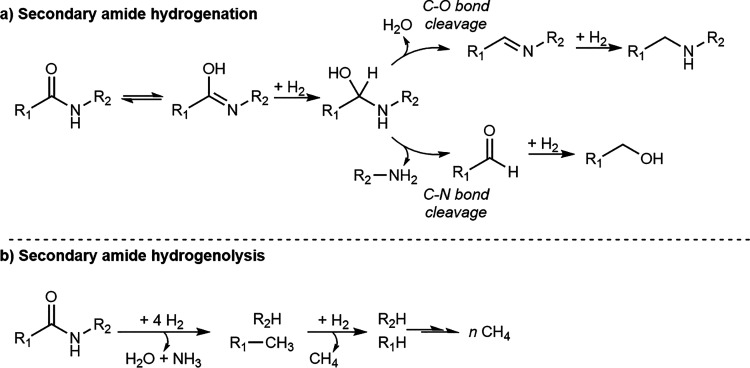
(a) Mechanism for the Hydrogenation of Secondary Amides, Showing the Competing C–N and C–O Bond Cleavage Pathways. (b) Reaction Products Typical for High Temperature Hydrogenolysis of Secondary Amides.

There is general consensus that the hydrogenation of a secondary amide begins with the formation of a hemiaminal intermediate (Scheme 1, hydrogenation pathway).^16^ This step is followed either by C–O bond cleavage, generating an imine and eliminating a water molecule, and subsequent hydrogenation of the imine to form a secondary amine, or by C–N bond cleavage, which results in the formation of a primary amine and an aldehyde, with the latter being hydrogenated to a primary alcohol. However, this mechanistic pathway is based on observations of amide reductions not employing molecular hydrogen,^17^ and has been validated only by theoretical calculations.^12,18^ There is a lack of direct experimental evidence for the formation of hemiaminal and imine/aldehyde intermediates during hydrogenation, as these species are extremely short-lived, especially under the harsh reaction conditions required in heterogeneous catalysis. Moreover, the mechanism for amide hydrogenation on heterogeneous catalysts is poorly understood compared to homogeneous metal complexes,^13,14^ due to the challenges of *operando* characterization of intermediates on solid-state catalysts. Almost all reported studies on amide hydrogenation have been conducted in a solvent, as the conditions required for solvent-free hydrogenation are typically too harsh to achieve high selectivity.^19^

At elevated temperatures, metal catalysts tend to cleave every C–heteroatom bond during the conversion of amides (Scheme 1, hydrogenolysis pathway).^20^ Previously, we reported the solvent-free hydrogenolysis of secondary amides into alkanes, water, and ammonia using a bifunctional Pt/CeO_2_ catalyst at 325 °C and under H_2_.^11^ Compared to a more active Ru/CeO_2_ catalyst, Pt/CeO_2_ exhibits excellent selectivity for hydrocarbon products, retaining all C–C bonds in the conversion of polyamides. However, the selectivity to amines or alcohols is low, even at lower reaction temperatures. Pt/CeO_2_ and Pt/C catalysts were compared for *N*-hexylhexanamide hydrogenation, with Pt/C showing lower activity but similar product selectivities (to alkanes) over longer reaction times. The majority of heterogeneous catalysts reported in literature favor C–O over C–N bond hydrogenation,^21−24^ as the former pathway can be promoted by carbonyl group adsorption on a Lewis acidic support.^24^ Notably, the competition between amide hydrogenation and hydrogenolysis pathways remains unclear, particularly under harsh, solvent-free conditions that are not readily accessible by other analytical techniques.

Recent advances in photoelectron photoionization coincidence (PEPICO) spectroscopy enable the detection of short-lived intermediates at low concentrations under *operando* conditions.^25,26^ Reactants, intermediates, and products emanating from the reactor are ionized if the photon energy exceeds their ionization energies, producing a photoelectron and a photoion, which are then detected in delayed coincidence.^27^ In addition, the detection of reactive radicals or intermediates can signal whether the reaction is confined on the catalyst surface or occurs (partially) in the gas phase.^28^ The photoionization spectrum is the *m*/*z* response of a neutral species as a function of photon energy and is often isomer-specific. In the photoion mass-selected threshold photoelectron spectrum (ms-TPES), the signal of the *m*/*z* peak of interest in coincidence with near-zero kinetic energy electrons, *i.e*. in threshold ionization, is considered. The ms-TPES often includes fingerprint regions with well-defined vibrational progressions for highly isomer-specific identification of intermediates and products as compared to the photoionization spectrum.^29^ This feature is particularly useful for observing isobaric reaction intermediates during amide hydrogenation, as compounds with identical mass-to-charge ratios (*m*/*z*), but different threshold photoelectron spectra, can be distinguished.

**Figure 1:**
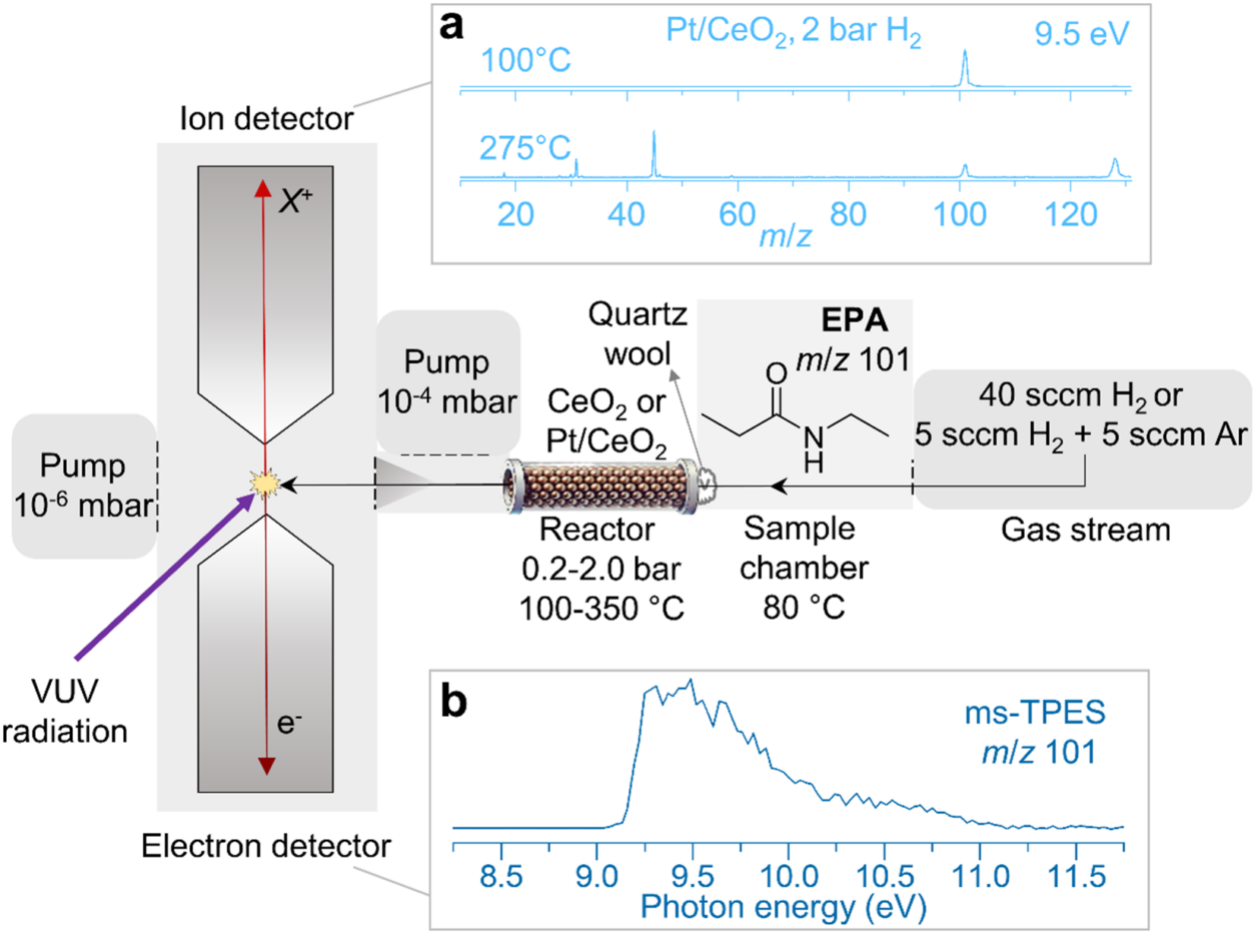
Experimental setup for *operando* photoelectron photoionization coincidence (PEPICO) spectroscopy. Detailed description of the experiments can be found in the Methods section. (a) Photoionization mass spectra were collected to record the *m*/*z* signal response at different photoionization energies, reaction temperatures, pressures, and catalysts. (b) The *m*/*z* 101 photoion mass-selected threshold photoelectron spectrum (ms-TPES) corresponds to the reactant *N*-ethylpropionamide (EPA). VUV – vacuum ultraviolet, sccm – standard cubic centimeters per minute.

Herein, the heterogeneously catalyzed hydrogenation of *N*-ethylpropionamide (EPA, *m*/*z* 101) was investigated under *operando* conditions via PEPICO spectroscopy (Figure 1). EPA is the smallest nonsymmetric secondary amide that includes C–C bonds in both R groups and is ideal to determine whether C–C bond hydrogenolysis occurs through the detection of methyl-derived products.^20^ In addition, the presence of nonsymmetric ethyl and propyl groups in EPA allows for the determination of whether an observed species originates from C–N alkylation or transamidation side reactions, which will generate secondary or tertiary products.^30^ By the detection of intermediates, *operando* PEPICO spectroscopy distinguishes amide hydrogenation and hydrogenolysis, providing insights into how the reaction mechanism determines selectivity, and from appropriate control experiments, delineates the roles of the Pt nanoparticles and CeO_2_ support. The focus was placed on investigating reactions which follow amide bond cleavage. PEPICO probes the gas phase, and strongly surface-bound reaction intermediates evade detection even if their lifetime is long.

## 2 Methods

### PEPICO Spectroscopy

The *operando* experiments were performed at the VUV beamline of the Swiss Light Source (SLS) using the Combustion Reaction Followed by double imaging Photoelectron Photoion Coincidence (CRF-PEPICO) spectrometer.^31^ A schematic for the reaction setup for the conversion of EPA is shown in Figure 1, and the detailed description of the end station can be found in ref (32). The catalyst was packed in a quartz microreactor, with a 200 or 50 μm nozzle diameter on the outlet. The pressure in the sample inlet system was 0.2 or 2 bar, which was saturated by EPA (TCI, 99.0%) or propionamide (TCI, 98.0%), which both have a vapor pressure of ca. 10 mbar at 80 °C.^33^ The gas stream, saturated with reactant, then entered the heated catalytic reactor with the catalyst bed kept in place with quartz wool on both sides before expanding into vacuum. The total gas flow rate over the catalyst was set to 10 sccm (standard cubic centimeters per minute) for the 0.2 bar conditions, consisting of 5 sccm H_2_ and 5 sccm Ar, while 40 sccm of H_2_ for the 2 bar conditions. This corresponds to a sample flux of 3.4 and 13.6 μmol/s, respectively. Assuming an average effective pressure of 0.1 vs 1 bar in the reactor, respectively, and a void fraction of 0.4, this flow rate corresponds to a flow velocity of 20 vs 8 m/s at room temperature, i.e., a backing gas residence time in the 3 cm long catalyst bed of 1 vs 2.5 ms at the representative temperature of 200 °C. When a product or intermediate desorbs from the catalyst, it enters the gas stream and forms a molecular beam after exiting the nozzle, which is skimmed as it enters the ionization chamber at ca. 10^–6^ mbar. Here, species are ionized by the monochromatic VUV synchrotron radiation, forming a photoion and a photoelectron, which are extracted in opposite directions by a 216 V/cm electric field. The charged particles are detected under velocity map imaging conditions in delayed coincidence to record the photoionization mass spectrum. A 5 min holding period preceded data acquisition at each temperature. While the sample flow was constant during the experiment, the velocities increase with temperature, which leads to lower effective densities in the ionization region, resulting in a slight signal decrease. Furthermore, as the expansion into vacuum gets more divergent with temperature, a smaller fraction of the flow arrives in the ionization region. Therefore, the signal intensities can only be compared qualitatively across temperatures, and not quantitatively. The mass spectral peak integrals at a single temperature only represent relative concentrations across the species qualitatively as the photoionization cross sections are in part unknown. The ms-TPES were obtained by scanning the photon energy, discriminating for close-to-zero kinetic energy electrons^34^ and plotting the threshold photoionization signal in the *m*/*z* ion channel of interest as a function of energy (see Supporting Methods S1.1 for details of ms-TPES data fitting). The TPES of EPA, propionamide, ethylpropylamine, methylpropionamide and propionitrile were obtained by measuring the pure compounds directly.

## 3 Results and Discussion

### 3.1 *Operando* PEPICO Spectroscopy Experiments with Pt/CeO_2_

The Pt/CeO_2_ catalyst investigated was synthesized following a literature procedure^11^ (see Supporting Methods S1.2 for synthesis procedures) and features well-dispersed Pt nanoparticles (∼2 nm) on a commercially available CeO_2_ support (∼25 nm) (see Figures S1–2 for electron microscopy images). PEPICO experiments were conducted at 2 and 0.2 bar H_2_. The lower pressure is expected to reduce the reaction rate, increasing the lifetime of reaction intermediates to accomplish isomer-selective detection. The pressure was controlled by adjusting the gas flow rate, using pure H_2_ for the reaction conducted under 2 bar and a 1:1 mixture of H_2_ and Ar for the reaction conducted under 0.2 bar pressure (referred to hereafter as 0.2 bar H_2_). The *m*/*z* peak integrals for species of interest were recorded at photon energies between their ionization onset and the onset of their dissociative photoionization, above which fragment ions at lower *m*/*z* are observed due to the loss of a neutral fragment. For instance, the measurements for EPA were recorded at 10 eV, only 0.5 eV above its calculated vertical ionization energy of 9.53 eV (cf. *m*/*z* 101 ms-TPES in Figure 1). To trace the production or depletion of a species, its peak integral was plotted as a function of increasing temperature starting at 100 °C, as the temperature of the sample chamber was set to 80 °C to volatilize EPA. To probe the stability of the *m*/*z* signals, the photoionization spectra obtained for the reactions under 2 bar H_2_ at 275 °C were taken 4 h apart from each other (Figure S3). The relative peak intensities of the *m*/*z* signals remained constant while the intensity of the reactant peak did not increase.

**Figure 2:**
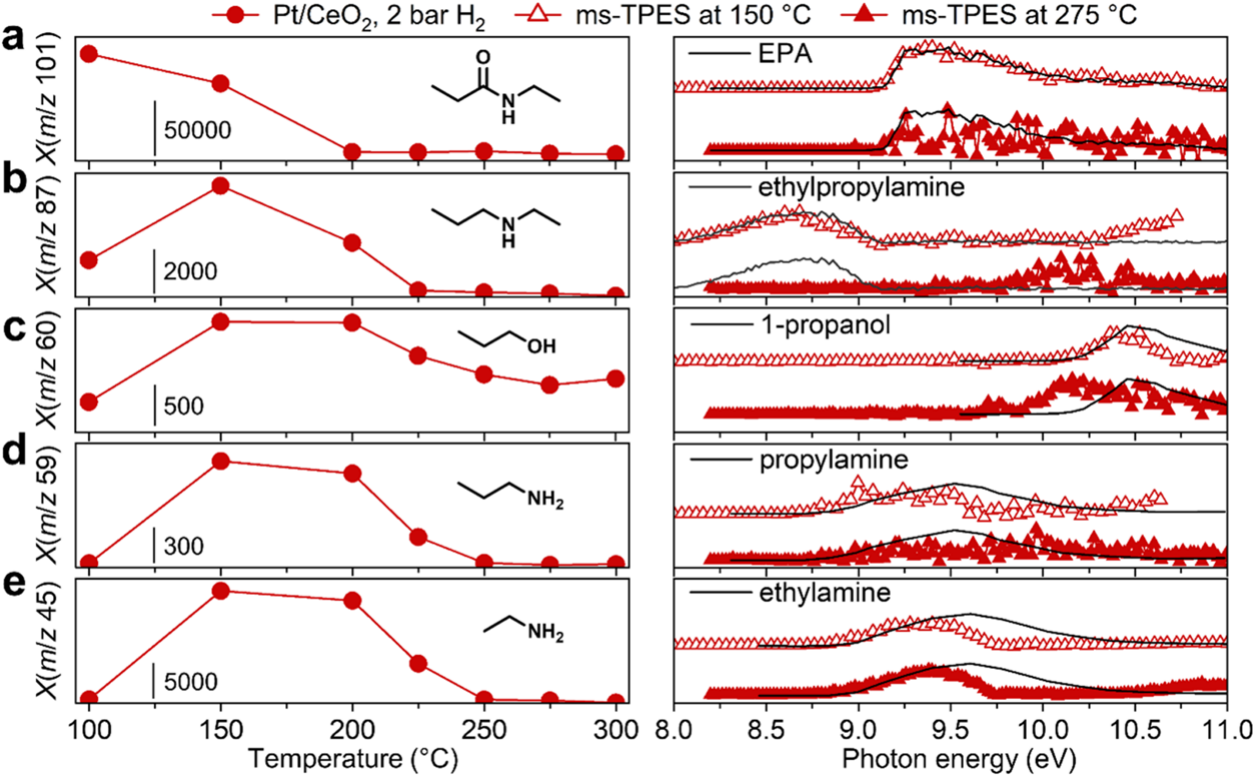
Temperature-dependent mass spectral peak intensities and ms-TPES for the conversion of EPA catalyzed by Pt/CeO_2_ under 2 bar H_2_, showing (a) the depletion of *m*/*z* 101 (EPA) and the formation of (b) *m*/*z* 87 (ethylpropylamine), (c) *m*/*z* 60 (1-propanol), (d) *m*/*z* 59 (propylamine), and (e) *m*/*z* 45 (ethylamine). The photoionization mass spectra were acquired at 9.5 eV for *m*/*z* 87, 59 and 45, 10 eV for *m*/*z* 101, and 11 eV for *m*/*z* 60. Reference spectra of EPA and ethylpropylamine were measured using pure samples in separate runs, whereas the photoelectron spectra of 1-propanol, propylamine and ethylamine were adapted from ref (49).

**Scheme 2:**
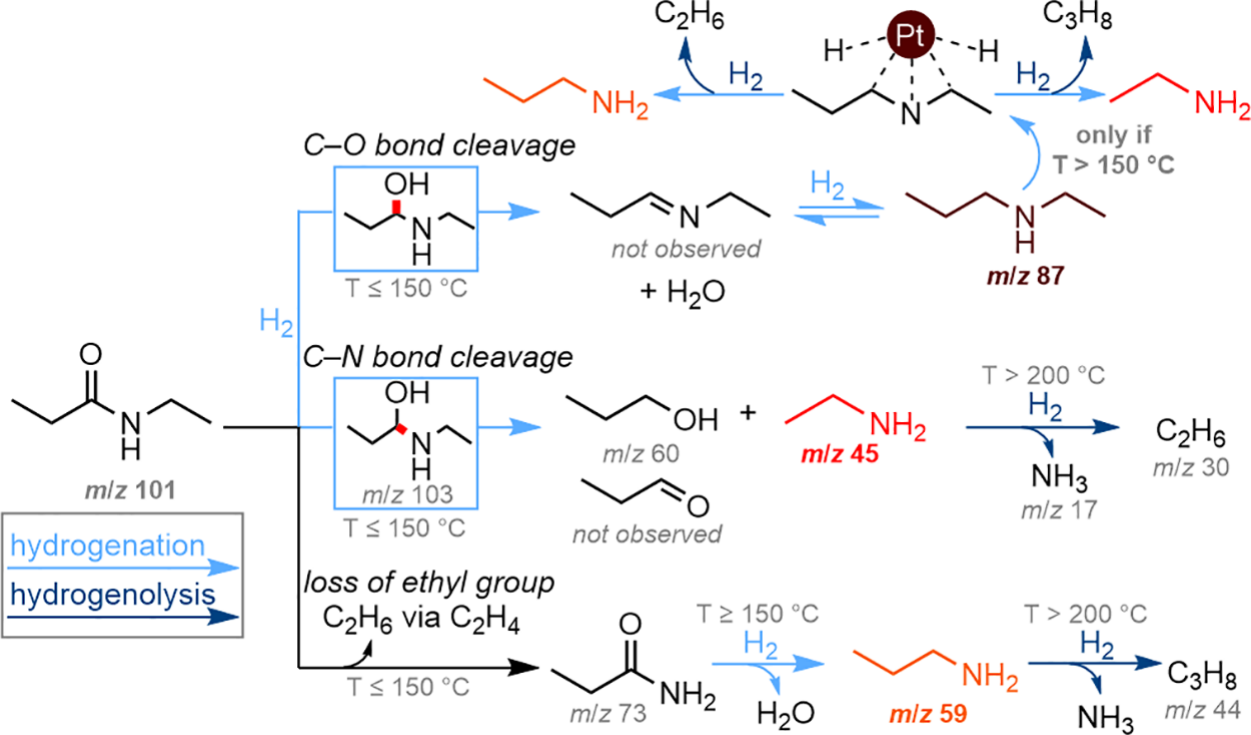
Observed Reaction Products from the Conversion of EPA Catalyzed by Pt/CeO_2_

As the reaction temperature increases, EPA is more readily depleted at 2 bar H_2_ (Figure 2a) than at 0.2 bar (Figure S4a). At 275 °C, EPA was only detected at 0.2 bar H_2_, indicating its rapid conversion under 2 bar of H_2_, and that the rate of amide bond activation on Pt/CeO_2_ is dependent on H_2_ pressure, as expected for hydrogenation reactions.^35^ To identify the reaction intermediates appearing under 2 bar H_2_, a ms-TPES scan was conducted at 150 °C, a temperature at which the reactant could still be detected. Under these conditions, the major peaks in the photoionization mass spectrum appear at *m*/*z* 87, 60, 59, and 45 (Figure S5) and were identified based on the reference ms-TPES as ethylpropylamine, 1-propanol, propylamine, and ethylamine, respectively (see Figure 2b–e and Scheme 2 for summary of reaction products). The primary amines were identified based on their ms-TPES up to 9.5 eV, above which dissociative ionization sets in and leads to the ms-TPES signals dropping to zero.^36^ Ethylpropylamine (*m*/*z* 87, Figure 2b) can be generated upon C–O bond cleavage of the hemiaminal intermediate (*m*/*z* 103) and subsequent hydrogenation of an ethylpropylimine intermediate. However, neither intermediate was positively identified from the ms-TPES, suggesting that these species are bound on the catalyst surface. The ethylpropylamine signal decreases above 150 °C, and is likely *de*hydrogenated at higher temperatures on the catalyst as the initiation step for amine C–N bond cleavage (Scheme 2, top pathway).^20^ Above 150 °C, ethylpropylamine generates either propane (*m*/*z* 44, Figure S6b) and ethylamine (*m*/*z* 45, Figure 2e), or ethane (*m*/*z* 30, Figure S6a) and propylamine (*m*/*z* 59, Figure 2d).

However, ethylamine, ethane, and propylamine were detected at 150 °C under 2 bar H_2_, whereas propane was not observed, although the two C–N bonds in ethylpropylamine are expected to undergo hydrogenation at comparable rates. These observations suggest that the primary amines do not originate from ethylpropylamine, nor does amine C–N bond cleavage occur at 150 °C. Instead, ethylamine may originate from C–N bond hydrogenation of EPA (Scheme 2, middle pathway), as its alcohol counterpart, 1-propanol (*m*/*z* 60, Figure 2c), was detected at 150 °C. Although propanal (*m*/*z* 58, Figure S7) was not observed in the ms-TPES, this may be due to its rapid hydrogenation to 1-propanol. The signal intensities for ethylamine and propylamine (Figure 2d–e) remain approximately constant between 150 and 200 °C, suggesting that the production and conversion of primary amines occur simultaneously at higher temperatures. Ethylpropylamine likely undergoes C–N bond hydrogenation between 150 and 200 °C to produce either ethylamine or propylamine, which then undergo hydrogenolysis to produce the respective alkane and ammonia (*m*/*z* 17, Figure S8). The ethylpropylamine signal was not observed above 200 °C, and the primary amines disappear from the spectra at slightly higher temperatures. These results indicate that selective hydrogenation of amides is only possible at temperatures below 150 °C on Pt/CeO_2_, as the amine products undergo further reactions at higher temperatures.

Due to the lower reaction rate at 0.2 bar H_2_, neither secondary nor primary amines were detected at 150 °C (Figure S9). Interestingly, while the signals for ethylpropylamine and propylamine were not observed at higher temperatures (Figure S4b and d), the signal intensity for ethylamine increased as the reaction temperature was raised from 150 to 250 °C (Figure S4e). This suggests that EPA can be selectively hydrogenated at the C–N bond at higher temperatures and subambient H_2_ pressures, which is not the typically favored pathway over Pt-based catalysts (Scheme 2, middle pathway).^24^ It is plausible that the CeO_2_ support promotes the amide C–N bond hydrogenation under these conditions instead of the Pt sites, which was subsequently confirmed by further experiments (see following section).

Notably, a peak was observed in the photoionization mass spectrum at *m*/*z* 73 at 275 °C for the reaction conducted under 0.2 bar H_2_ (Figure S10a). This peak was also present under 2 bar H_2_ but with a much lower intensity (Figure S10b). The ms-TPES identified the peak at *m*/*z* 73 as propionamide (Figure S11), a primary amide generated formally by ethyl loss from EPA. Propionamide was therefore likely being produced and immediately hydrogenated to propylamine under 2 bar H_2_, accounting for the propylamine previously observed at 150 °C (Scheme 2, bottom pathway). It is unlikely that the Pt nanoparticles catalyze the direct conversion of a secondary amide to a primary amide, particularly under hydrogenation conditions that favor amide reduction. Hence, the generation of propionamide is the second process where the CeO_2_ support appears to be responsible. Further insights into the formation of propionamide in the presence of CeO_2_ are described in the next section.

### 3.2 *Operando* PEPICO Spectroscopy Experiments with CeO_2_

**Figure 3:**
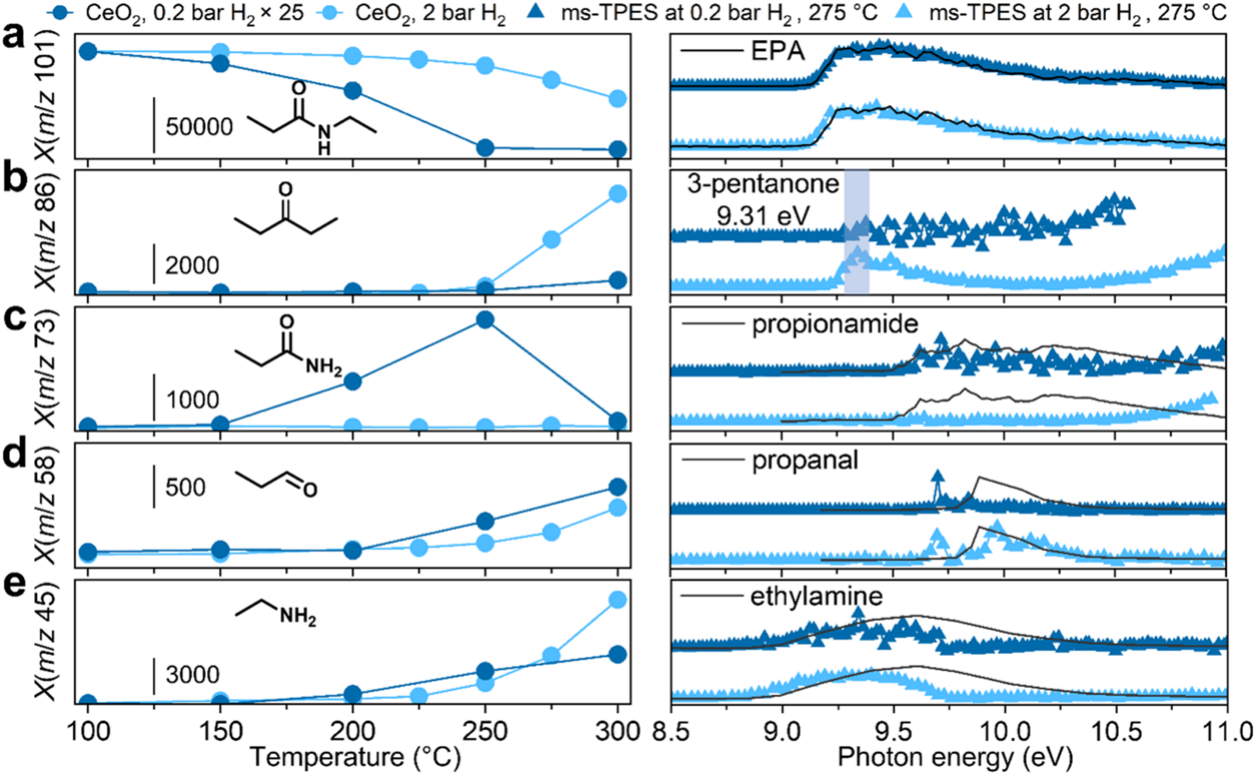
Temperature-dependent mass spectral peak intensities and ms-TPES for the conversion of EPA catalyzed by CeO_2_, showing (a) the depletion of *m*/*z* 101 (EPA) and the formation of (b) *m*/*z* 86 (3-pentanone, as identified from its ionization energy), (c) *m*/*z* 73 (propionamide), (d) *m*/*z* 58 (propanal) and (e) *m*/*z* 45 (ethylamine). The photoionization mass spectra were acquired at 9.5 eV for *m*/*z* 86 and 45, 10 eV for *m*/*z* 101 and 58, and 10.5 eV for *m*/*z* 73, and the signal intensities were multiplied by 25 for the case of 0.2 bar H_2_. Reference spectra of EPA and propionamide were measured using pure samples in separate runs. The reference photoelectron spectra of propanal and ethylamine, and the ionization energy of 3-pentanone were adapted from refs (49) and (50), respectively.

CeO_2_ is inactive for the conversion of EPA below 150 °C regardless of the H_2_ pressure. The metal oxide support has numerous Lewis acid sites with strong redox capabilities, and has been reported to exhibit activity in dehydration and dehydrogenation reactions at high temperatures.^37^ Indeed, the signal intensity of EPA was also observed to decrease above 150 °C (Figure 3a). Furthermore, the equilibrium between EPA and the major reaction products, i.e., propionamide (*m*/*z* 73, Figure 3c) and ethylene (*m*/*z* 28), is also shifted to the products at low pressure. Under 0.2 bar, the signal intensity of propionamide reached a maximum at 250 °C before decreasing, due to deamination to yield ammonia (Figure S12) and propanal (*m*/*z* 58, Figure 3d).^38^ The absence of propylamine (Figure S13) reveals that the hydrogenation of propionamide is not promoted by CeO_2_.

Although propionamide was not observed in the photoionization mass spectra under 2 bar H_2_ (Figure S14), its counterpart ethylene was observed (Figure S15), suggesting that propionamide is rapidly deaminated under these conditions. At 0.2 bar H_2_, ethylene likely remains adsorbed on the CeO_2_ surface, explaining its absence in the ms-TPES, and acts presumably as a coke precursor,^39^ also evidenced by a notably darker discoloration observed on the spent CeO_2_ after the reaction was conducted under 0.2 bar compared to 2 bar (Figure S16). Furthermore, a distinct blue color, which rapidly faded upon exposure to air, was observed on the spent CeO_2_ catalyst under 2 bar H_2_ when removed from the reactor. This color change was attributed to the partial reduction of CeO_2_ to CeO_2–*x*_ during the reaction, suggesting that it has significantly more surface oxygen vacancies under 2 bar H_2_.^40^

**Scheme 3:**
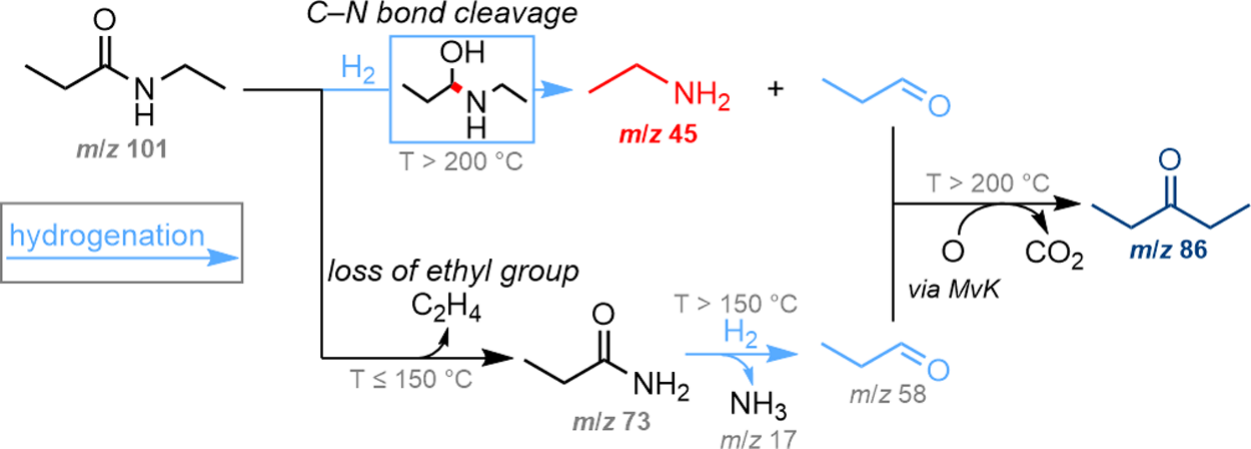
Observed Reaction Products from the Conversion of EPA Catalyzed by CeO_2_ MvK – Mars–van Krevelen mechanism.

Interestingly, hydrogenation of the C–N bond to ethylamine (*m*/*z* 45, Figure 3e) was observed on CeO_2_ above 200 °C and under both H_2_ pressures (Figure 3e, Scheme 3 top pathway). This activity may be attributed to the Lewis acid sites on CeO_2_, implying that the C–N bond hydrogenation activity previously observed under 0.2 bar H_2_ with Pt/CeO_2_ also originates from the support rather than the Pt nanoparticles. CeO_2_ is an effective catalyst for the dehydrogenation of alcohols,^41^ hence it is unlikely to hydrogenate propanal to 1-propanol. Therefore, it is not surprising that, although the signal intensity for ethylamine increases with reaction temperature, the intensity for 1-propanol remains barely detectable, and was absent in the ms-TPES at 275 °C (Figure S17). This suggests that ethylamine and propanal are produced from C–N bond cleavage. Although propanal is the favored product from both possible EPA conversion pathways using CeO_2_ (Scheme 3), it can undergo further reaction to produce 3-pentanone (*m*/*z* 86, Figure 3b).^42^ The formation of 3-pentanone results from the aldol addition of two equivalents of propanal, followed by dehydrogenation and the elimination of CO_2_, where the additional oxygen atom may originate from the CeO_2_ lattice via a Mars–van Krevelen mechanism (MvK).^42,43^ The major product obtained from the conversion of EPA catalyzed by CeO_2_ was 3-pentanone, which had a greater signal intensity under 2 bar H_2_ compared to 0.2 bar, due to the relatively larger amount of propanal being formed. These results suggest that CeO_2_ is more active under 2 bar H_2_, presumably due to the higher degree of support reduction to generate more surface oxygen vacancies.

Although the Lewis acidity of CeO_2_ assists C–N bond cleavage in EPA, it may also hinder the conversion by promoting undesirable transamidation reactions at higher temperatures.^44^ Minor transamidation products were observed from the CeO_2_-catalyzed reactions, including *N*-methylpropionamide (*m*/*z* 87, Figure S18). Based on the ms-TPES analysis, ethylpropylamine (*m*/*z* 87) was excluded as the spectral carrier. *N*-methylpropionamide is suspected to be a product of C_1_ coupling with propionamide. However, the methyl source likely originates from CO_2_ rather than being produced from C–C bond cleavage, given the poor hydrogenolysis ability of CeO_2_. This was supported by the negligible change in methane (*m*/*z* 16) signal intensity observed between 100 to 350 °C (Supporting Figure 19). In addition, *N*,*N*-diethylpropionamide (*m*/*z* 129, Figure S20), another transamidation side product, was observed in the reactions conducted under 2 bar H_2_ with both CeO_2_ and Pt/CeO_2_. However, the signal intensity of *N*,*N*-diethylpropionamide is significantly higher over Pt/CeO_2_ than over CeO_2_, as greater quantities of ethylamine are generated in the Pt/CeO_2_-catalyzed reactions.

### 3.3 *Operando* PEPICO Spectroscopy Experiments for the Conversion of Propionamide

**Figure 4:**
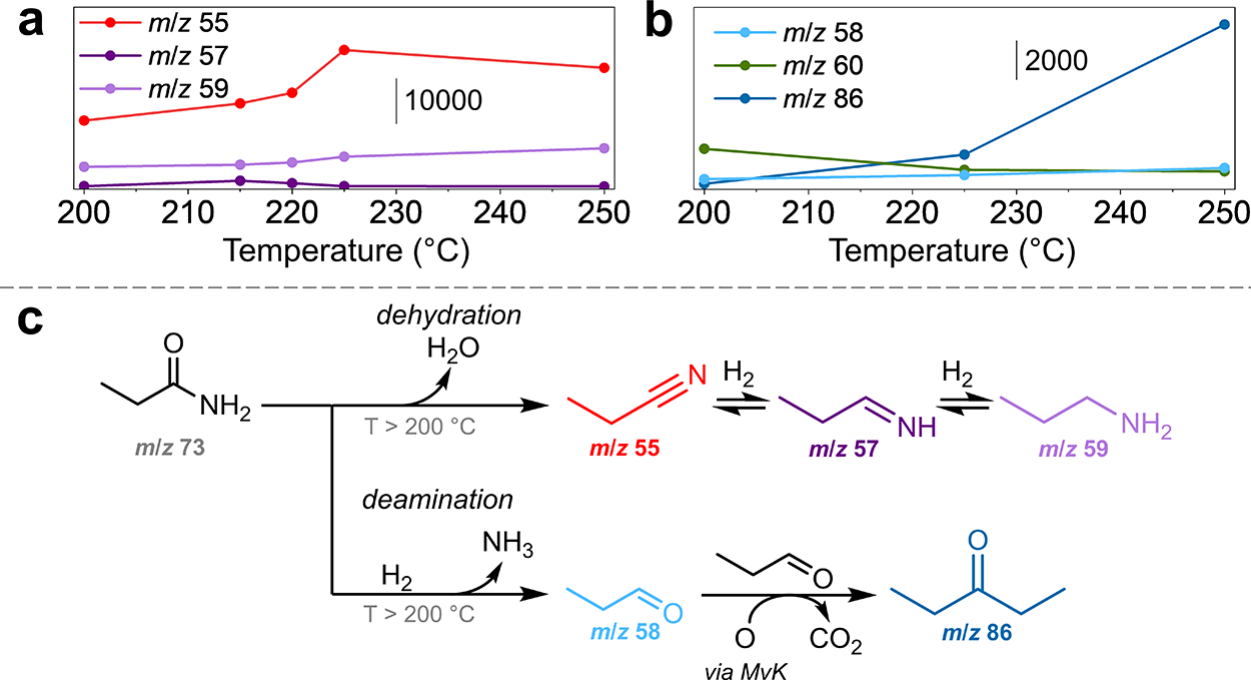
Relative intensities of the major product peaks detected in the photoionization mass spectra between 200 and 250 °C for the conversion of propionamide, with (a) Pt/CeO_2_ and (b) CeO_2_, under 2 bar H_2_. The peak intensities were acquired at 9.5 eV for *m*/*z* 59 (propylamine) and *m*/*z* 86 (3-pentanone), 10 eV for *m*/*z* 58 (propanal), 11 eV for *m*/*z* 57 (propylimine) and *m*/*z* 60 (1-propanol), and 12.5 eV for *m*/*z* 55(propionitrile). (c) Observed reaction products from the conversion of propionamide catalyzed by Pt/CeO_2_ (top pathway) and CeO_2_ (bottom pathway). MvK – Mars–van Krevelen mechanism.

As propionamide was generated from EPA in the presence CeO_2_ and considered to be an important intermediate, *operando* experiments were performed using propionamide as the reactant under 2 bar H_2_. The reactant signal was only observed at the higher reactor temperature of 200 °C at the start of the experiment, which is likely due to the stronger adsorption of the primary amide on the catalysts. Hence, 200 °C was used as the onset reaction temperature allowing comparison of the relative product selectivities from the conversion of propionamide using both Pt/CeO_2_ and CeO_2_. The major peak observed from the Pt/CeO_2_-catalyzed reaction is at *m*/*z* 55 (Figure 4a), identified as propionitrile based on ms-TPES analysis (Figure S21). Propionitrile can be generated by dehydration of propionamide, a reaction promoted by Pt,^45^ which has been reported as the initial step in the hydrogenation of primary amides.^16^ However, although the subsequent single hydrogenation intermediate 1-propylimine (*m*/*z* 57) was detected in trace amounts, its signal intensity began to decrease much earlier than that of propionitrile, at 215 °C. The signal intensity of the complete hydrogenation product, propylamine (*m*/*z* 59), increases up to 250 °C, suggesting that the 1-propylimine intermediate was rapidly converted to propylamine at temperatures above 215 °C. The rate of increase in the propylamine signal with increasing temperature was small, likely because it undergoes hydrogenolysis to propane and ammonia under these conditions. From the higher signal intensities of unsaturated products, such as propionitrile in the conversion of propionamide or *m*/*z* 85 in the conversion of EPA, Pt/CeO_2_ favors the dehydrogenation of amines at higher temperatures, even under 2 bar H_2_ pressure.

The CeO_2_ support has been reported to favor the hydration of nitriles to amides,^46^ hence accounting for the negligible propionitrile signal in the CeO_2_-catalyzed reaction (Figure 4b). Notably, the major product generated was 3-pentanone (*m*/*z* 86), which is consistent with the results obtained using EPA as the reactant. Furthermore, the intensity of propanal (*m*/*z* 58) was observed to plateau with increasing reaction temperature, whereas the intensity for 1-propanol (*m*/*z* 60) decreases. This suggests that propanal is produced directly from the deamination of propionamide, at approximately twice the rate at which it was converted into 3-pentanone. Hence, the conversion of the primary amide proceeds rapidly on CeO_2_ at higher pressures, which explains why propionamide was not detected during the conversion of EPA under 2 bar H_2_. The observations from the *operando* experiments reveal that the Lewis acidic support promotes dehydrogenation and aldol addition of the alcohol product during amide hydrogenation, thus hindering selectivity in the Pt/CeO_2_-catalyzed reactions.

### 3.4 Reaction Energy Computations

**Figure 5:**
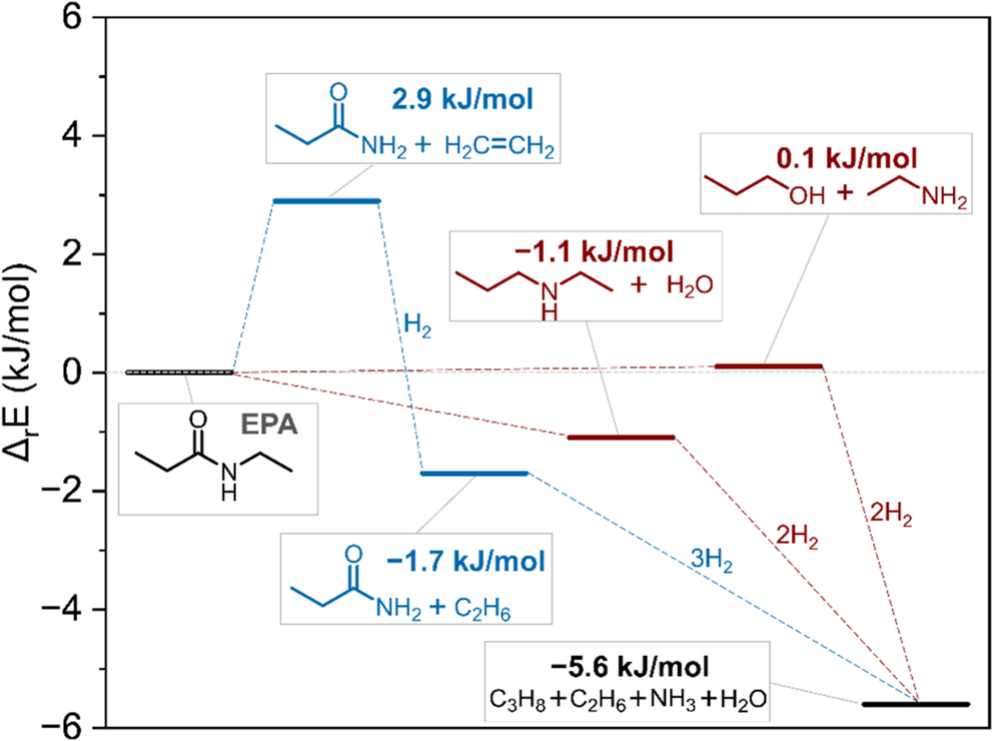
Favorability of different conversion pathways for EPA based on the computed reaction energies at 0 K. The formation energies for each species are provided in Table S1.

The reaction energies to the relevant products (Δ_r_*E*) were computed to assess the thermodynamic feasibility of EPA conversion to propionamide (Figure 5). CBS-QB3 calculations (see Supporting Methods S1.3 for calculations) reveal that the cleavage of the ethyl–N bond to form ethylene and propionamide is endothermic by Δ_r_*E* = 2.9 kJ/mol, which is only slightly less energetically favorable than the amide hydrogenation pathways corresponding to full cleavage of the C–N bond (Δ_r_*E* = 0.1 kJ/mol) or the C–O bond (Δ_r_*E* = −1.1 kJ/mol). The process becomes slightly more exothermic if ethylene is further hydrogenated to ethane (Δ_r_*E* = −1.7 kJ/mol), which is feasible with a hydrogenation catalyst such as Pt/CeO_2_. Furthermore, when comparing the free energies of formation at 298 K (Figure S22), the difference between the amide-to-amide pathway and the amide-to-amine/alcohol pathways becomes even smaller. These results demonstrate a negligible energy difference between the reaction pathways during amide hydrogenation, suggesting that product selectivities are controlled by the activation energies (kinetic control), which in turn depend on the catalyst.

### 3.5 Mechanism for the Conversion of EPA with Pt/CeO_2_ and CeO_2_

**Scheme 4:**
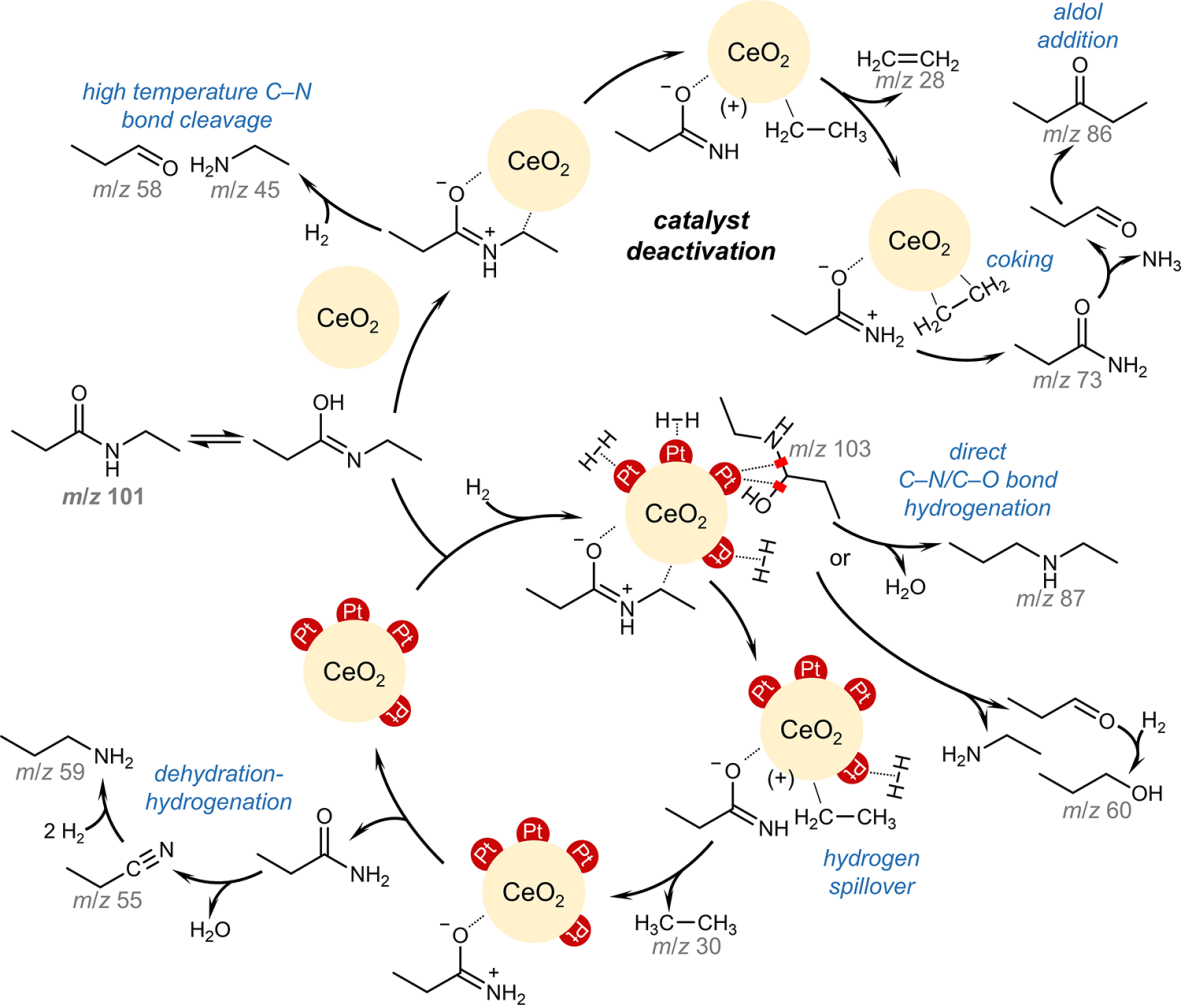
Proposed Mechanism Showing the Various Reaction Pathways during the Conversion of EPA on Pt/CeO_2_ and CeO_2_ under a Hydrogen Atmosphere The (+) positive charge on CeO_2_ represents an oxidation of Ce^3+^ to Ce^4+^.

Since the PEPICO results are qualitative, the reaction was performed under batch conditions for a quantitative analysis of amide hydrogenation over Pt/CeO_2_. The solvent-free conversion of *N*-dodecylhexanamide was performed in 75 mL autoclaves for 24 h under 5 bar H_2_ (Table S2, see Supporting Methods S1.4 for reaction procedure). The results are consistent with PEPICO spectroscopy, with Pt/CeO_2_ favoring amines as the major hydrogenation products. However, the higher yields of secondary/tertiary amines and amides suggest that the batch reactions favor the polyalkylation and transamidation products, as previously observed in heterogeneously catalyzed amide hydrogenation.^3,12,23^ Based on these results, we propose a tentative reaction mechanism for the conversion of secondary amides (using EPA as an example) catalyzed by both Pt/CeO_2_ and CeO_2_ (Scheme 4). For clarity, the hydrogenolysis and alkylation products are not included in the scheme. First, the adsorption of EPA (*m*/*z* 101) occurs at the Lewis acid sites on CeO_2_, where the carbonyl group may be activated through a zwitterionic resonance structure.^24^ If a proximal Pt site is present, the adsorbed EPA will be hydrogenated to the hemiaminal intermediate (*m*/*z* 103), before undergoing C–O bond cleavage to generate water and ethylpropylamine (*m*/*z* 87). The previously proposed hemiaminal and ethylpropylimine (*m*/*z* 85) intermediates could not be detected in the gas phase, suggesting that these intermediates are extremely short-lived or entirely surface-bound. At higher temperatures, C–N bond cleavage is facilitated by both Pt-sites and CeO_2_ Lewis acid sites to generate ethylamine (*m*/*z* 45) and propanal (*m*/*z* 58), which will be hydrogenated by the Pt nanoparticles to 1-propanol (*m*/*z* 60).

Following the upper pathway shown in Scheme 4, CeO_2_-adsorbed EPA can undergo cleavage of the ethyl–N bond, producing a surface-bound primary amide and ethyl-derived species. These surface species may be radicals, as CeO_2_ is an excellent radical scavenger due to its redox properties.^47,48^ Surface amide can abstract a hydrogen atom from the ethyl-derived species to generate propionamide (*m*/*z* 73), which then undergoes deamidation on CeO_2_ to produce propanal, which is finally converted to 3-pentanone (*m*/*z* 86) via aldol addition. The ethyl group may form ethylene (*m*/*z* 28) after hydrogen transfer or remain chemisorbed, leading to coking and catalyst deactivation. Following the lower pathway, on Pt/CeO_2_ the propionamide is dehydrated (Scheme 4, bottom) to propionitrile (*m*/*z* 55), followed by subsequent hydrogenation to propylamine (*m*/*z* 59). In addition, hydrogen spillover from the Pt nanoparticles facilitates the hydrogenation of the ethyl-derived species to ethane, therefore preventing coke formation on Pt/CeO_2_. This mechanism demonstrates the active roles played by both the metal and support during the hydrogenation of EPA. CeO_2_ promotes the adsorption of the amide on the catalyst, whereas the Pt nanoparticles promote the transfer of hydrogen to the amide. Synergistic effects were evidenced from the bifunctionality of the catalyst, such as the ability of the Pt nanoparticles to prevent coke formation and the high capability of CeO_2_ to cleave C–N bonds at higher temperatures. However, side reactions that hinder the selectivity to hydrogenation products also occur, such as the Pt nanoparticles promoting amine hydrogenolysis and CeO_2_ facilitating conversion of alcohol products.

## 4 Conclusions

The conversion of the secondary amide, *N*-ethylpropylamide (EPA), on the bifunctional Pt/CeO_2_ catalyst has been scrutinized with *operando* PEPICO spectroscopy. The catalyst is selective for amide hydrogenation to produce amines at temperatures up to 150 °C, after which the formation of side products begins to increase. At temperatures above 200 °C, non-hydrogenation products include ethane, propane and ammonia, produced in hydrogenolysis reactions promoted by the Pt nanoparticles, as well as 3-pentanone, produced through aldol addition catalyzed by CeO_2_. The absence of gaseous radicals and reactive intermediates suggests that the process is surface-confined, which supports the central role of the catalyst in driving selectivities. In future, the high time resolution of PEPICO could be capitalized in isotope switching experiments to gain insights into the surface kinetics.

Although both the metal and the support are essential for the reaction—due to the excellent hydrogen transfer capability of the Pt nanoparticles and the oxophilicity of CeO_2_—side reactions significantly hinder the selectivity toward hydrogenation products, such as secondary amines. To achieve better selectivity, heterogeneous amide hydrogenation catalysts should focus on strategies to mitigate side reactions catalyzed by the support material while preserving the beneficial synergies the support provides. Moreover, the revealed mechanisms illustrate that selective amide hydrogenation is inherently challenging over strongly hydrogenating metal surfaces. These insights should pave the way for the rational design of more selective catalysts.

## Supporting Information Available

Additional methods, catalyst characterization, and experimental data (PDF)
