# Supplementary material for: Unveiling the Roles of Pt and CeO2 during Solvent-Free Amide Hydrogenation Utilizing Operando Photoelectron Photoionization Coincidence Spectroscopy
Source: ACS Catal. 2025 Apr 2;15(8):6216–25. doi: 10.1021/acscatal.4c07955 (PMC12012760; doi:10.1021/acscatal.4c07955)
Supplement: Supplementary file 1 — cs4c07955_si_001.pdf [file cs4c07955_si_001.pdf]

Title

Unveiling the roles of Pt and CeO_2 during solvent-free amide hydrogenation utilizing *operando* photoelectron photoionization coincidence spectroscopy

Author list

Xinbang Wu[a], Rosie J. Somerville[a], Andras Bodi[b]*, Roland C. Turnell-Ritson[a], Zihao Zhang[b], Jan Romano de Gea[a], Jaques-Christopher Schmidt[a], Patrick Hemberger[b]*, Paul J. Dyson[a]*

*corresponding authors

Affiliations

a Institute of Chemical Sciences and Engineering, École Polytechnique Fédérale de Lausanne (EPFL), Lausanne, Switzerland

b Laboratory of Synchrotron Radiation and Femtochemistry, Paul Scherrer Institute, Villigen, Switzerland

S1. Supplementary Methods

S1.1 ms-TPES data fitting

Isomer-selective detection was performed using ms-TPES by comparing the obtained spectra with reference spectra to accurately identify the neutral spectral carrier.[1] In some of the plots, the absence of a spectral carrier was evident, leading to poor agreement with the reference spectra or a completely missing ms-TPES signal for the target neutral (Figure S4, S13). The signal-to-noise ratio of the experimental ms-TPES depends on the intensity of the corresponding *m*/*z* peak in the photoionization mass spectrum. The experimental and reference spectra are overlaid by normalizing both spectra to their maxima when the experimental spectrum shows non-zero intensity in the energy range of interest. The measured species can be accurately assigned to the correct isomer, if the ms-TPES and the reference photoelectron spectrum agree well. However, it is noteworthy that dissociative photoionization will lead to a decrease in the ms-TPES signal, as the molecule begins fragmenting and its ms-TPES contribution shows up in a lower *m*/*z* channel. For instance, ethylamine can be conclusively identified based on the upper traces, although the experimental *m*/*z* 45 ms-TPES vanishes at 9.75 eV as the ethylamine cation fragments and loses hydrogen.

S1.2 Synthesis of Pt/CeO_2

K_2PtCl_4 (PMO, 213 g, 10 wt.% Pt relative to CeO_2) was dissolved in ultrapure water (50 mL) and mixed with CeO_2 (Sigma-Aldrich, 1 g). The mixture was stirred for 3 hours and then the

water was removed on a rotary evaporator. The catalyst was calcined at 300 °C in argon, redispersed in water to remove excess Pt salts, and then filtered and calcinated at 550 °C in air. See Figures S1 and S2 for electron microscopy images of the resulting $PtCeO_2$ catalyst.

S1.3 Quantum chemical calculations

Reaction energy calculations were carried out using the CBS-QB3 composite method[2,3] as implemented in the Gaussian 16 suite of programs.[4] The optimized Cartesian coordinates in Å of EPA, propionamide, and *N*-ethylpropylamine, for which the minimum-energy conformer is not trivially known, are given below:

EPA

C	-3.03490	0.19052	-0.07554
H	-3.13325	0.33009	-1.15506
H	-3.04137	1.17777	0.38718
H	-3.90332	-0.37162	0.27589
C	-1.73682	-0.54033	0.26879
H	-1.70533	-0.75377	1.34425
H	-1.68498	-1.50768	-0.24213
C	-0.49826	0.29751	-0.04902
O	-0.49621	1.51448	0.03599
N	0.60581	-0.41993	-0.41789
H	0.54493	-1.42582	-0.42018
C	1.90541	0.19902	-0.65034
H	1.70726	1.24443	-0.88783
H	2.35841	-0.26541	-1.53171
C	2.84775	0.09928	0.55183
H	3.03941	-0.94333	0.82313
H	3.80892	0.56927	0.32435
H	2.41657	0.60551	1.41831

Propionamide

C	-1.66616	0.31745	-0.13100
H	-1.69819	0.60855	-1.18342
H	-1.71938	1.23072	0.46234
H	-2.54570	-0.29481	0.08182
C	-0.38355	-0.44772	0.18805
H	-0.40611	-0.80747	1.22430
H	-0.28981	-1.33796	-0.44328
C	0.86780	0.41909	0.04396
O	0.83981	1.63436	0.07814
N	2.03404	-0.27825	-0.11409
H	2.89888	0.23868	-0.12570
H	2.06839	-1.28262	-0.08113

N-ethylpropylamine

C	3.15046	-0.30885	-0.07713
H	3.16103	-0.91906	-0.98313
H	3.23958	-0.98063	0.78314
H	4.02960	0.34082	-0.08124
C	1.86503	0.50989	-0.00327
H	1.87866	1.14167	0.90444

H	1.82012	1.19817	-0.85498
C	-0.58094	0.36550	0.01072
H	-0.62486	1.06073	-0.83664
H	-0.66345	0.98589	0.92382
C	-1.77024	-0.59222	-0.06119
H	-1.71799	-1.29142	0.78324
H	-1.67143	-1.19602	-0.96858
C	-3.12132	0.12706	-0.04303
H	-3.21930	0.80594	-0.89582
H	-3.94968	-0.58443	-0.09036
H	-3.24601	0.72041	0.86813
N	0.68792	-0.35342	-0.06795
H	0.73280	-1.03004	0.68982

S1.4 Batch experiments

10 mg of catalyst and 100 mg of *N*-dodecylhexanamide were added to a 20 mL glass vial containing a stirring bar, which was then placed inside a 75 mL autoclave. The autoclave was sealed, pressurized with 5 bar H_2 (or N_2) after three purging cycles, and inserted in a heating jacket. After 24 hours, the autoclave was removed from the heating jacket and placed in a water bath to stop the reaction. The autoclave was depressurized, and the products from the glass vial were extracted with diethyl ether. The yields of the products (shown in Table S2) were obtained from gas chromatography–mass spectrometry (GC-MS) analysis using *p*-xylene (30 mg) as an internal standard.

S2. Supporting Tables

Table S1. CBS-QB3 calculations of different pathways for the hydrogenation or dissociation of EPA.

Molecule	Molar mass (g/mol)	E_{0K} (E_h)	G_{298K} (E_h)
N-ethylpropylamide	101	-326.56129	-326.59616
ethylpropylamine	87	-252.56751	-252.60006
propionamide	73	-248.11469	-248.14489
propanol	60	-193.99541	-194.02326
ethylamine	45	-134.89669	-134.92219
propane	44	-118.85587	-118.88091
ethane	30	-79.63057	-79.65200
ethylene	28	-78.41664	-78.437511
water	18	-76.33748	-76.35514
ammonia	17	-56.5	-56.47823
hydrogen	2	-1.16609	-1.17758

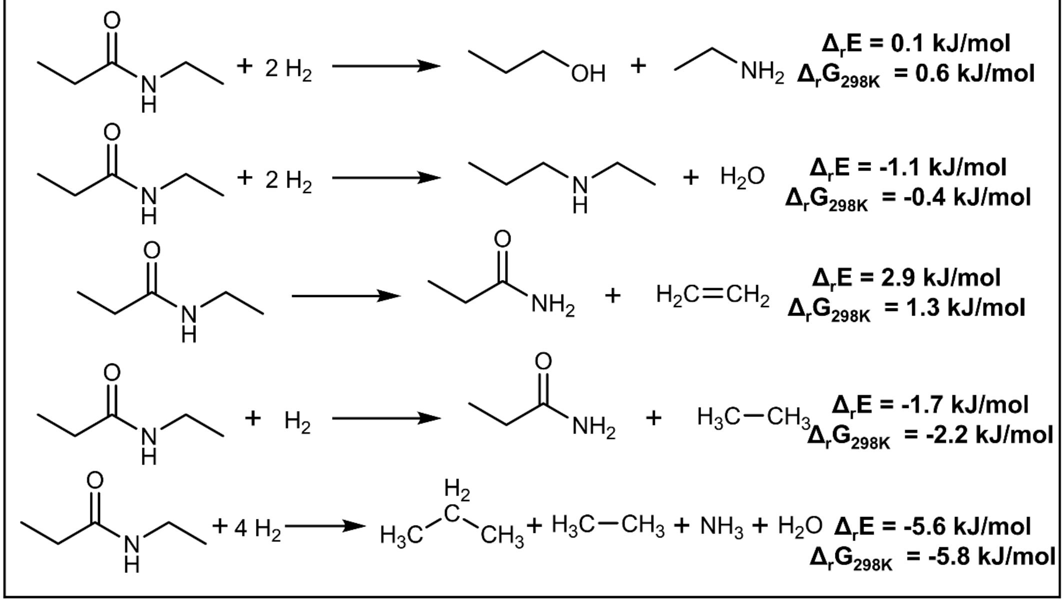

Table S2. Batch reaction of *N*-dodecylhexanamide using Pt/CeO_2 and CeO_2, analyzed via GC-MS.

Entry	Catalyst	Temperature (°C)	**1** (%)	**2** (%)	**3** (%)	**4** (%)	**5** (%)	**6** (%)	**7** (%)	**8** (%)	**9** (%)	**10** (%)	**11** (%)
1	CeO_2	250	0.4	0.4	0	0.4	0	0	0	0	5.4	0.5	1.2
2	CeO_2[a]	250	0.4	0.3	0.1	0.3	0	0	0	0	0.8	0.4	1.2
3	CeO_2	325	1.0	3.8	3.2	3.8	0	0	0	1.3	4.0	2.1	2.2
4	$PtCeO_2$	250	0.1	2.6	0	0	3.2	0.8	1.2	2.3	5.7	2.4	1.4
5	$PtCeO_2$	325	1.1	2.7	0	0.4	34.9	12.1	1.3	0.6	9.6	3.1	6.1
6	-	250	0.2	0.1	0	0	0	0	0	0	0.2	0.2	0.5
7	-	325	0.1	0.3	0.3	0	0	0	0.2	0	0.2	0.4	0.2

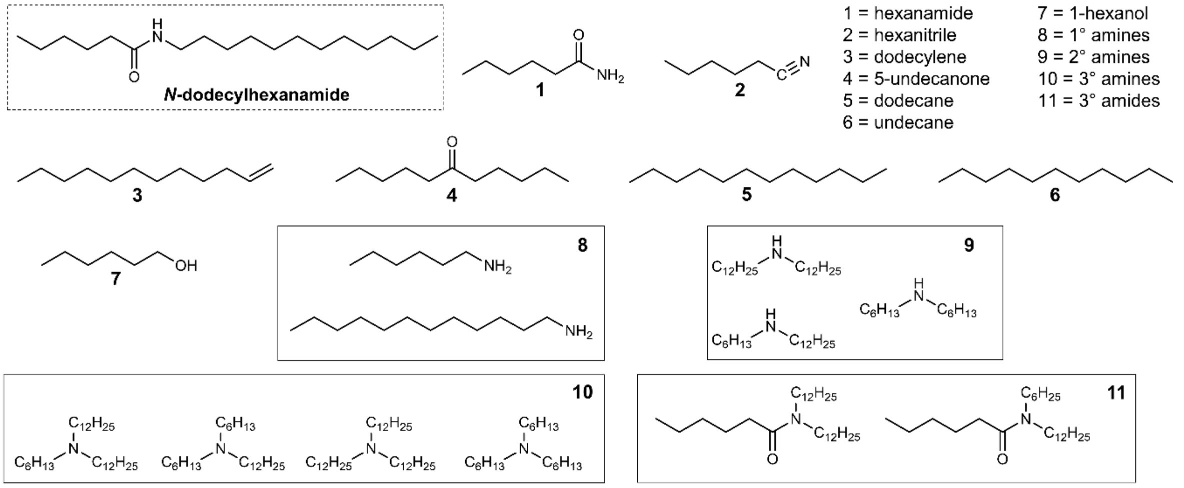

The yields of 1-7 were obtained from calibration curves of pure compounds, whereas the combined yields of 8-11 were calculated from their effective carbon numbers. *N*-hexane and *n*-pentane were also detected as alkane products but were not quantified as they overlap with the solvent signal. Reaction conditions: 10 mg catalyst and 100 mg reactant under 5 bar H_2, [a] 5 bar N_2.

S3. Supporting Figures

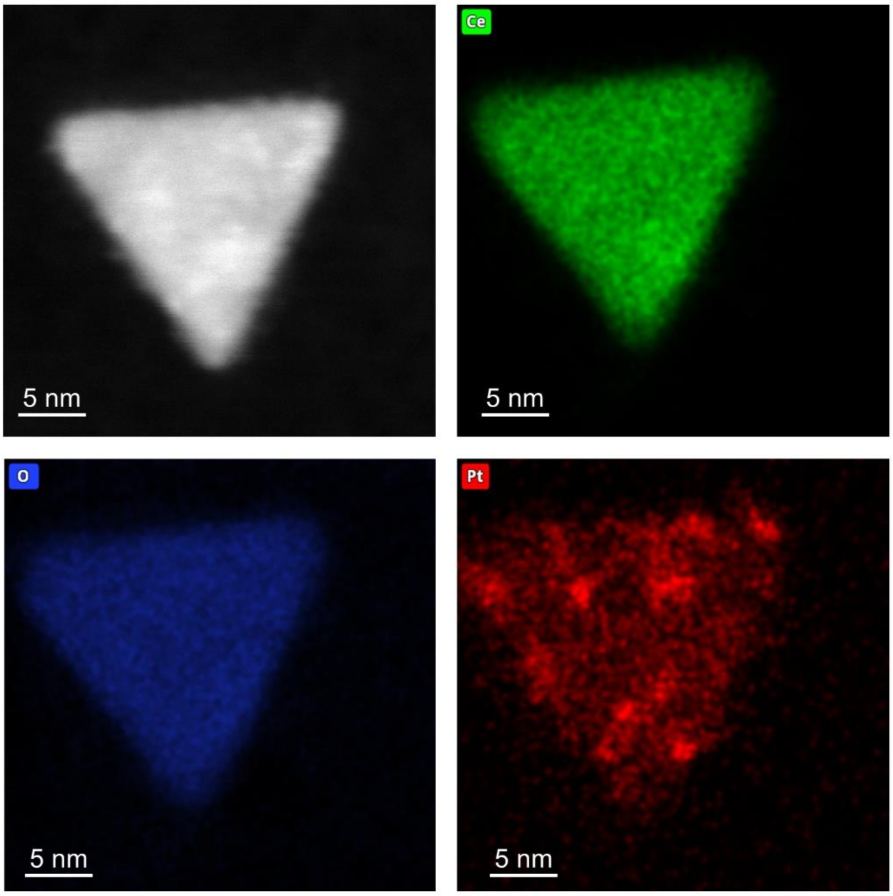

Figure S1. Scanning transmission electron microscopy (STEM) image and energy-dispersive X-ray spectroscopy (EDX) mapping of Pt/CeO_2.

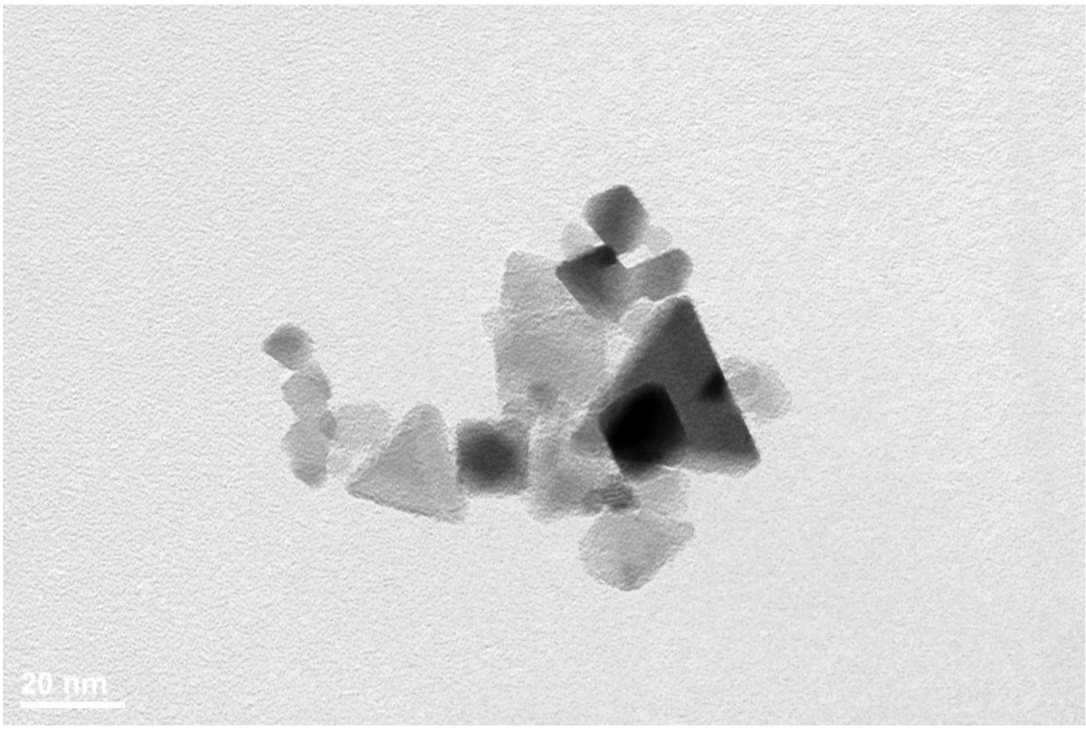

Figure S2. Bright field TEM image of the CeO_2 support.

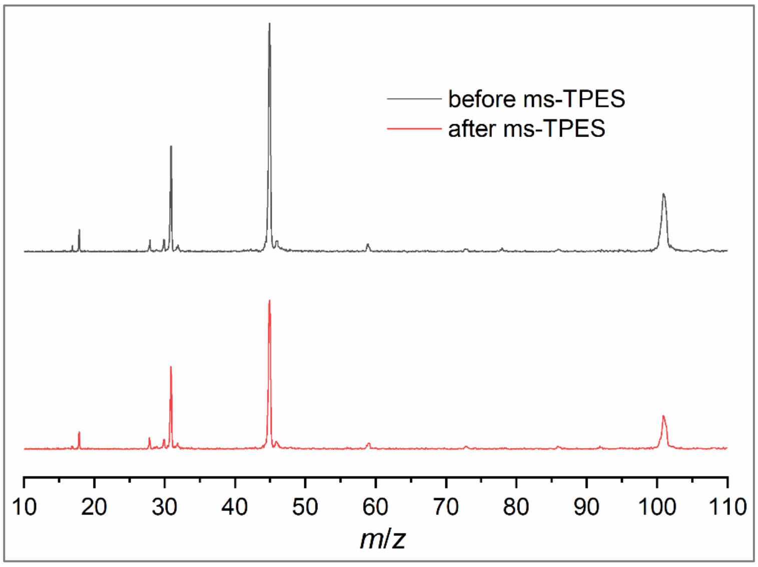

Figure S3. Photoionization spectra for the Pt/CeO_2-catalysed conversion of EPA (at 9.5 eV) under 2 bar H_2 at 275 °C. (a) before ms-TPES acquisition, (b) after 4 hours of ms-TPES acquisition.

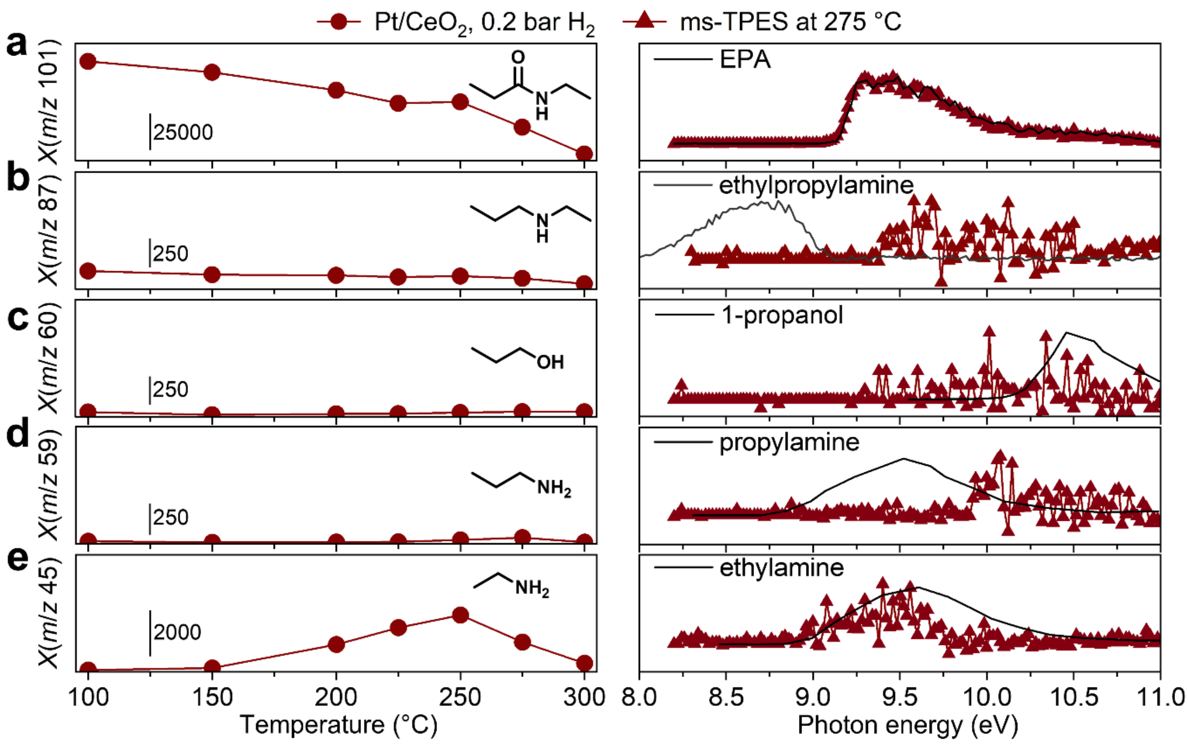

Figure S4. Temperature-dependent mass spectral peak intensities and ms-TPES for the conversion of EPA catalyzed by Pt/CeO_2 under 0.2 bar H_2, showing (a) the depletion of *m*/*z* 101 (EPA) and the absence of (b) *m*/*z* 87 (ethylpropylamine), (c) *m*/*z* 60 (1-propanol), (d) *m*/*z* 59 (propylamine), and the formation of (e) *m*/*z* 45 (ethylamine). The photoionization mass spectra were acquired at 9.5 eV for *m*/*z* 87, 59 and 45, 10 eV for *m*/*z* 101, and 11 eV for *m*/*z* 60. Reference spectra of EPA and ethylpropylamine were measured using pure samples in separate runs, whereas the photoelectron spectra of 1-propanol, propylamine and ethylamine were adapted from ref. 5.

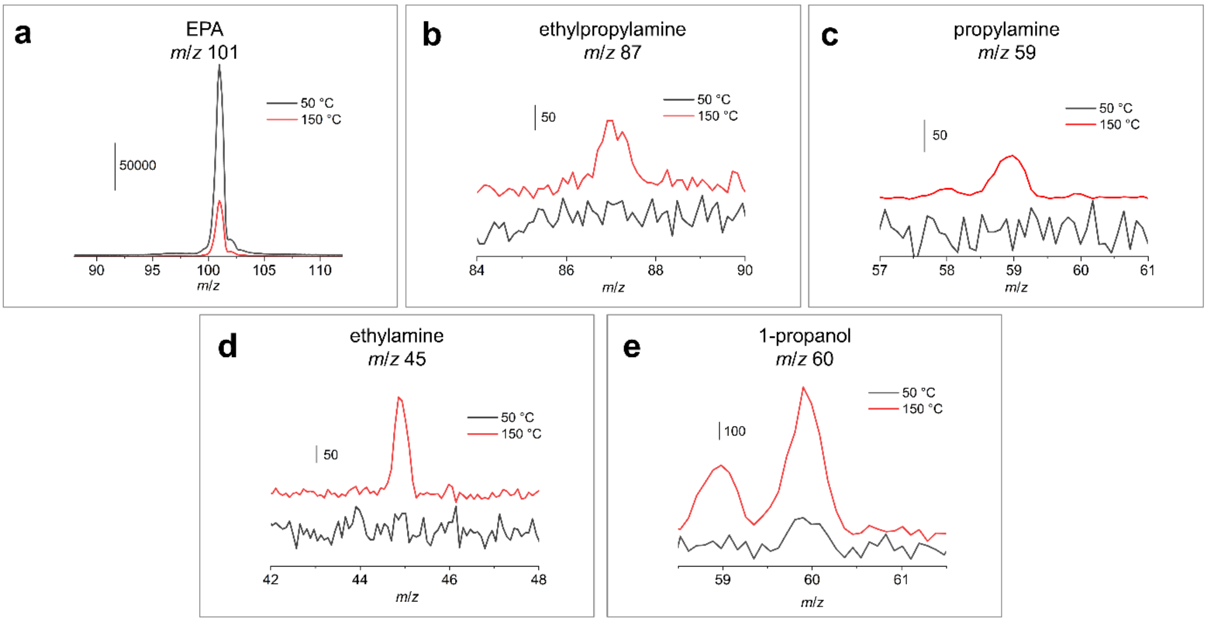

Figure S5. Photoionization mass spectra during the conversion of EPA catalyzed by Pt/CeO_2 under 2 bar H_2, acquired at (a) 10 eV, (b-d) 9.5 eV, and (e) 11 eV.

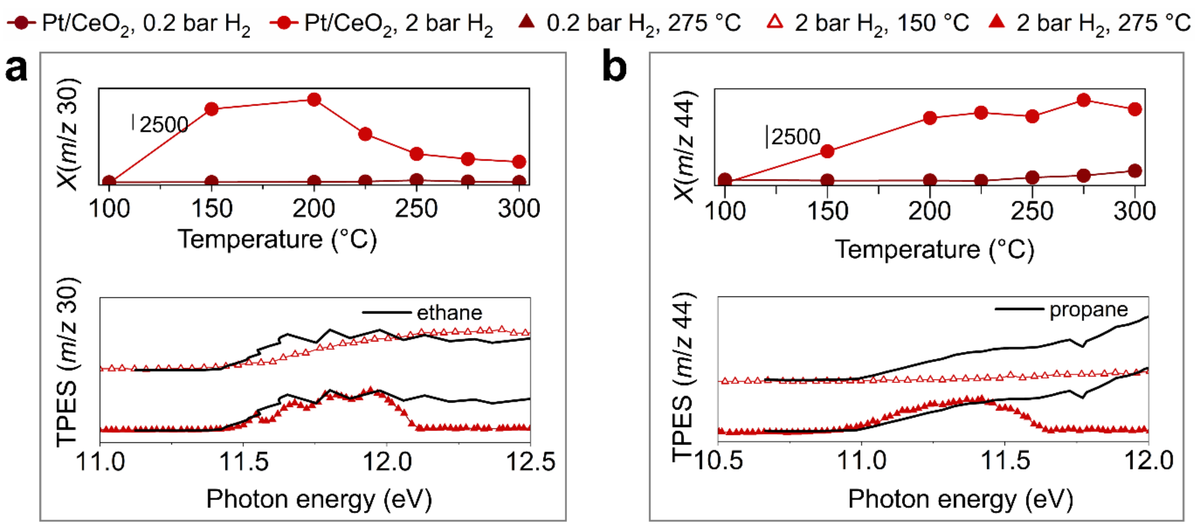

Figure S6. Temperature-dependent peak intensities and ms-TPES showing the formation of a) *m/z* 30 and b) *m/z* 44 during the conversion of EPA catalyzed by Pt/CeO_2. The photoionization mass spectra were acquired at 12.5 eV. Dissociative ionization of unconverted EPA has contributed to the *m/z* 30 signal, especially at lower temperatures with less conversion (as observed from ms-TPES of *m/z* 30 at 150 °C). The photoelectron spectra of ethane and propane were adapted from ref. 6.

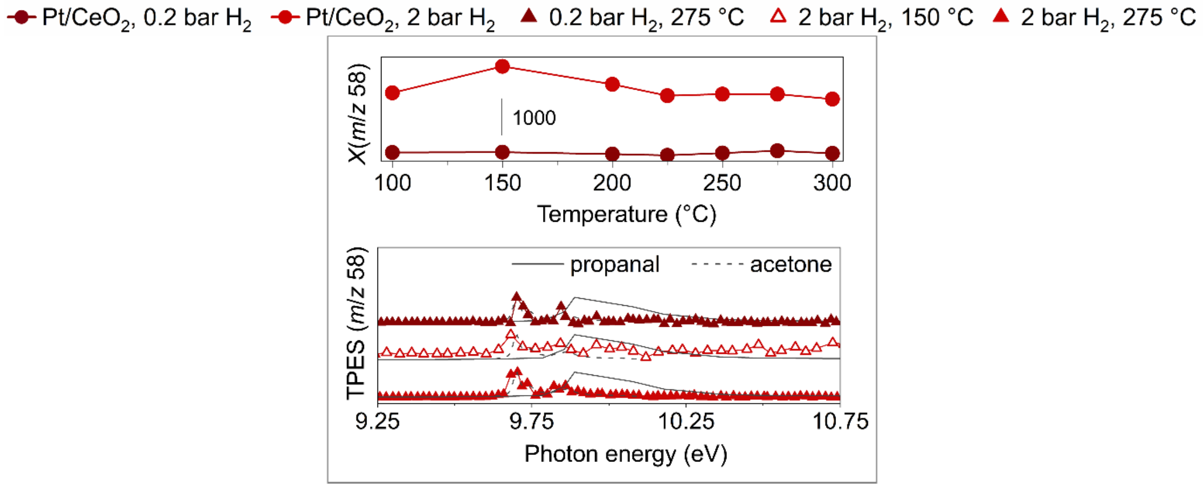

Figure S7. Temperature-dependent peak intensities and ms-TPES showing the absence of *m*/*z* 58 during the conversion of EPA catalyzed by Pt/CeO_2. The photoionization mass spectra were acquired at 10 eV. The reference photoelectron spectra of propanal and acetone were adapted from refs. 5 and 7, respectively. Acetone was identified as a contaminant in the chamber from previous measurements.

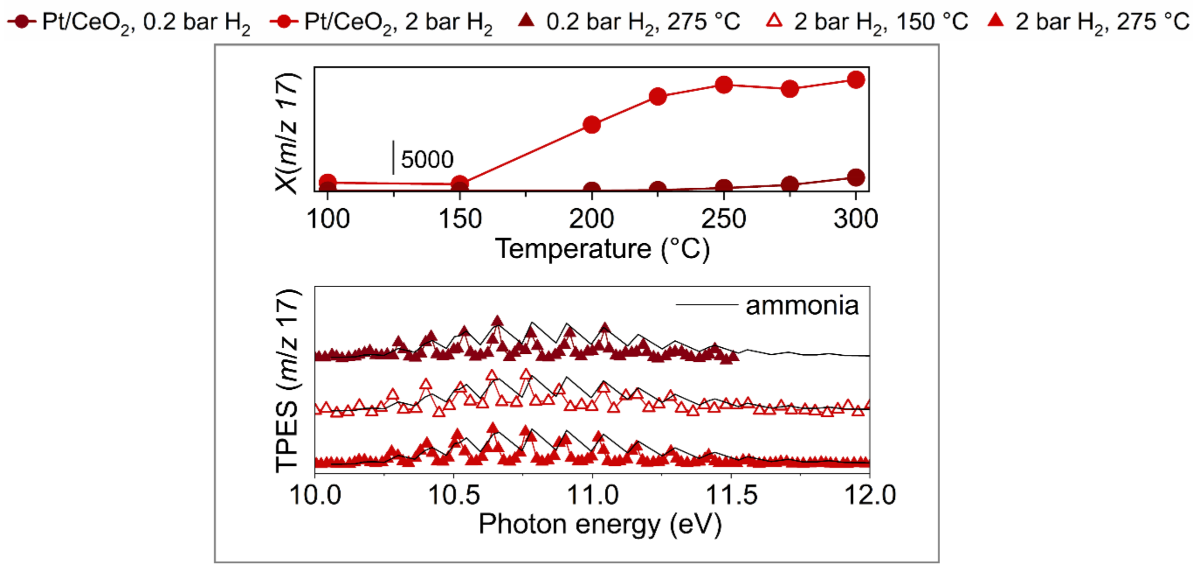

Figure S8. Temperature-dependent peak intensities and ms-TPES showing the formation of *m*/*z* 17 during the conversion of EPA catalyzed by Pt/CeO_2. The photoionization mass spectra were acquired at 11 eV and the photoelectron spectrum of ammonia was adapted from ref. 8.

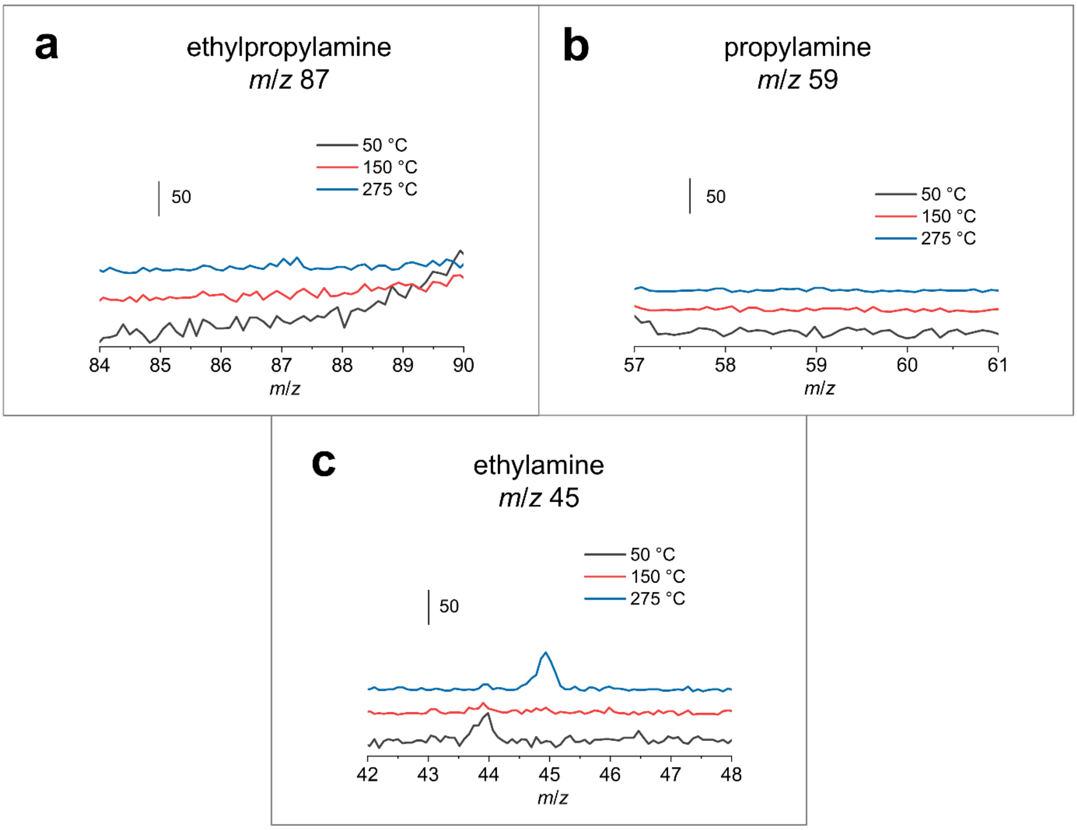

Figure S9. Photoionization mass spectra for the conversion of EPA catalyzed by Pt/CeO_2 under 0.2 bar H_2, acquired at a-c) 9.5 eV.

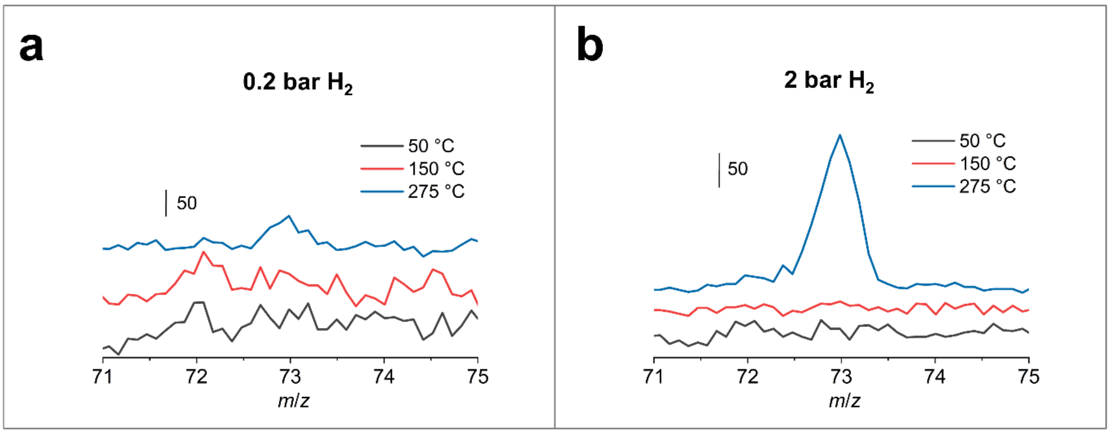

Figure S10. Photoionization mass spectra for the conversion of EPA catalyzed by Pt/CeO_2 under a) 2 bar H_2, and b) 0.2 bar H_2, acquired at 10.5 eV.

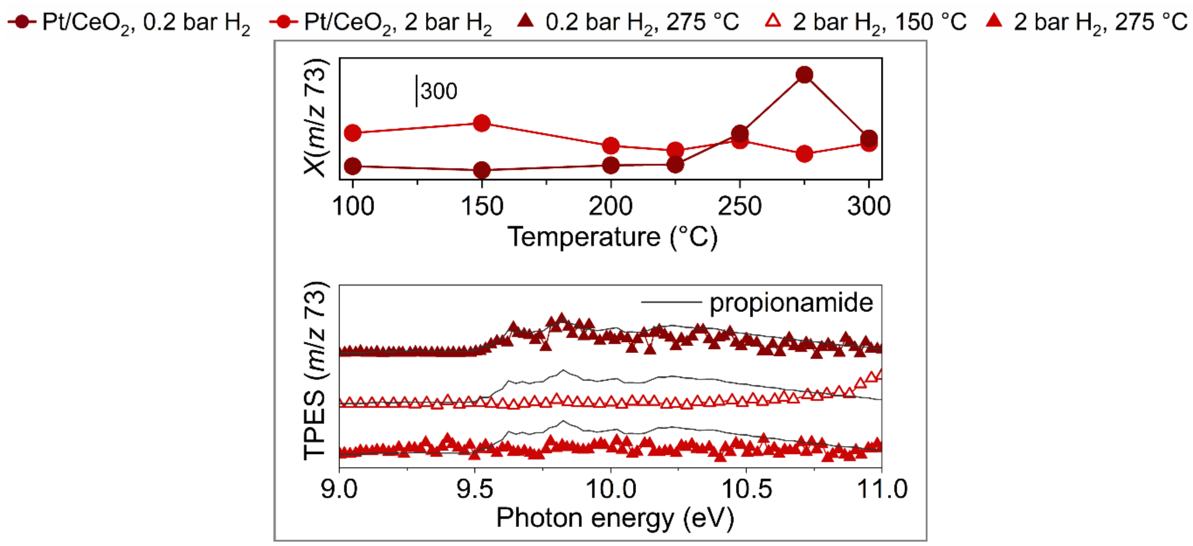

Figure S11. Temperature-dependent peak intensities and ms-TPES showing the formation of *m*/*z* 73 during the conversion of EPA catalyzed by Pt/CeO_2. The photoionization mass spectra were acquired at 10.5 eV. Reference spectra of propionamide was measured using pure samples in separate runs.

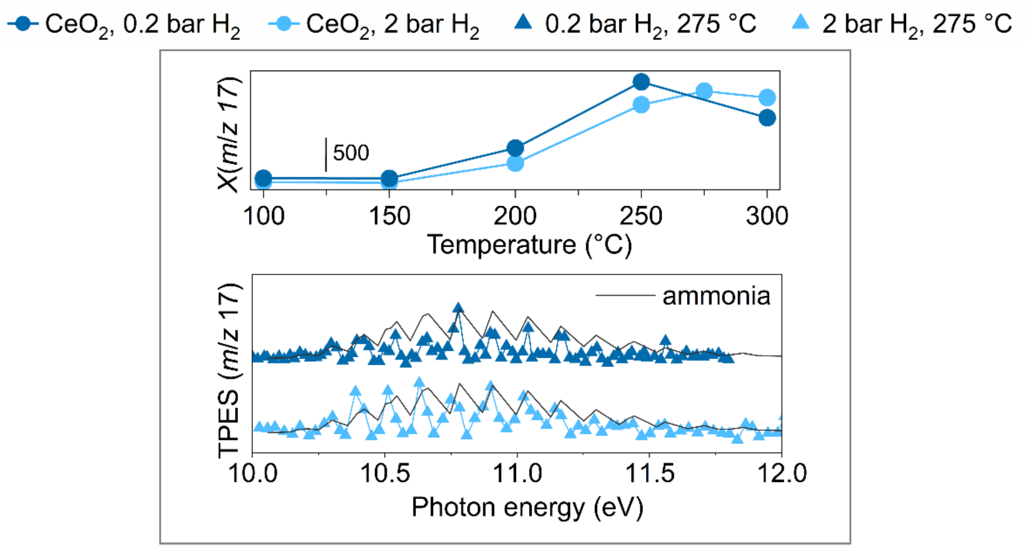

Figure S12. Temperature-dependent peak intensities and ms-TPES showing the formation of *m*/*z* 17 during the conversion of EPA catalyzed by CeO_2. The photoionization mass spectra were acquired at 11 eV and the reference photoelectron spectrum of ammonia was adapted from ref. 8.

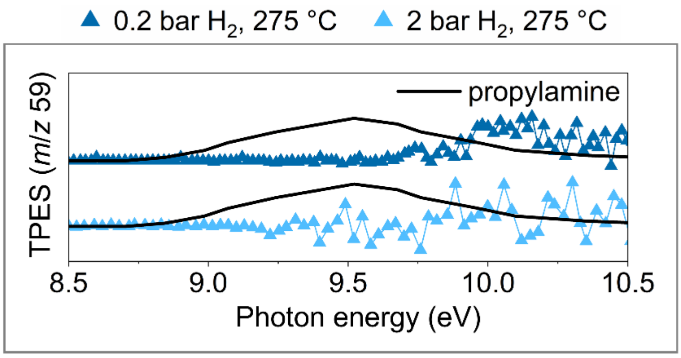

Figure S13. ms-TPES showing the absence of *m/z* 59 during the conversion of EPA catalyzed by CeO_2. The reference photoelectron spectrum of propylamine was adapted from ref. 5.

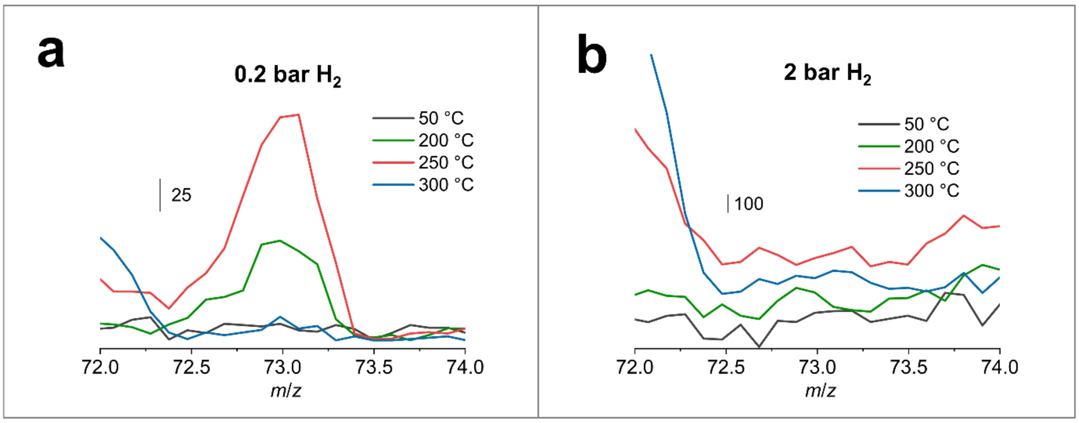

Figure S14. Photoionization mass spectra for the conversion of EPA catalyzed by CeO_2 under a) 0.2 bar H_2, and b) 2 bar H_2, acquired at 10.5 eV. The peak at *m/z* 72 is a dissociative ionization product of EPA.

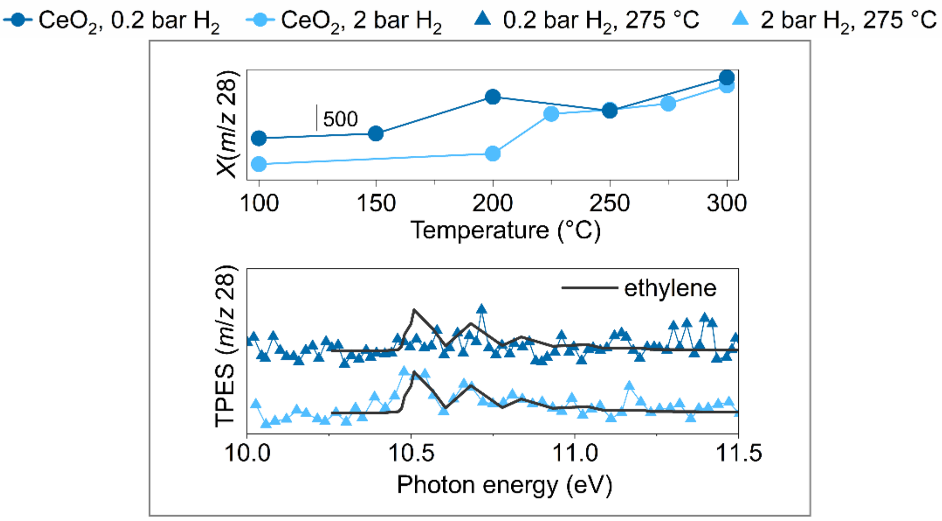

Figure S15. Temperature-dependent peak intensities and ms-TPES showing the formation of *m*/*z* 28 during the conversion of EPA catalyzed by CeO_2. The photoionization mass spectra were acquired at 11 eV and the reference photoelectron spectrum of ethylene was adapted from ref. 5.

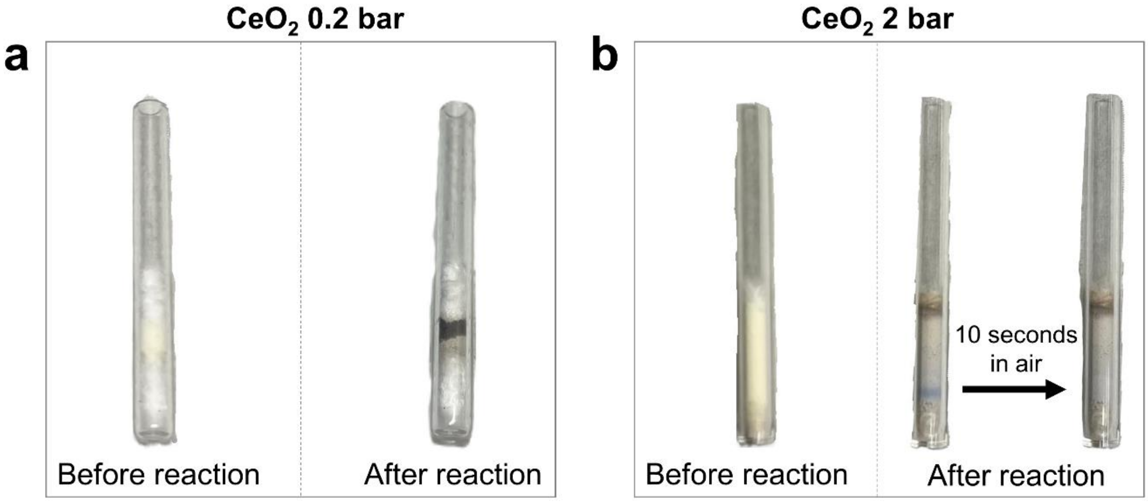

Figure S16. Images of the quartz reactor chamber containing fresh and spent CeO_2 catalyst for the reactions conducted under a) 0.2 bar and b) 2 bar H_2.

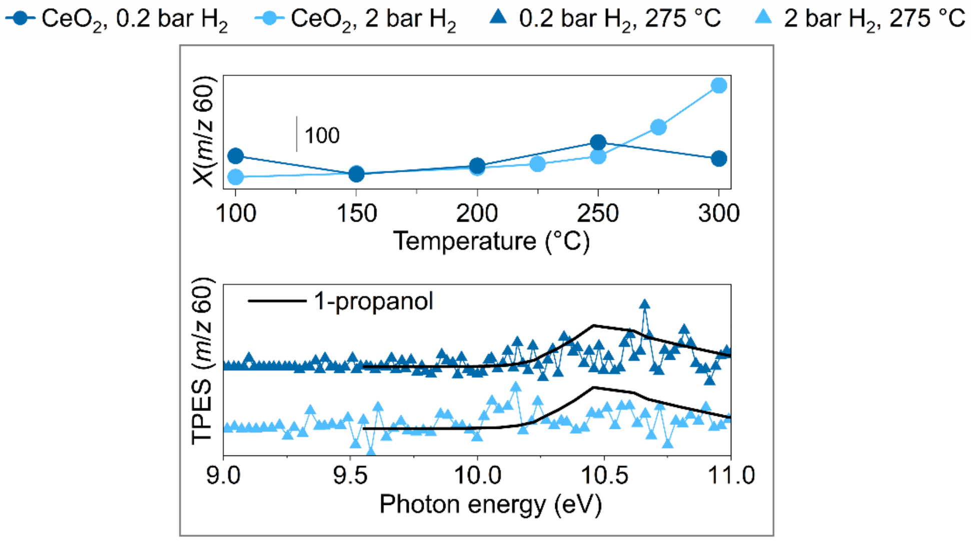

Figure S17. Temperature-dependent peak intensities and ms-TPES for the conversion of EPA catalyzed by CeO_2, showing the absence of *m*/*z* 60. The photoionization mass spectra were acquired at 11 eV. The reference photoelectron spectrum for 1-propanol was adapted from ref. 5.

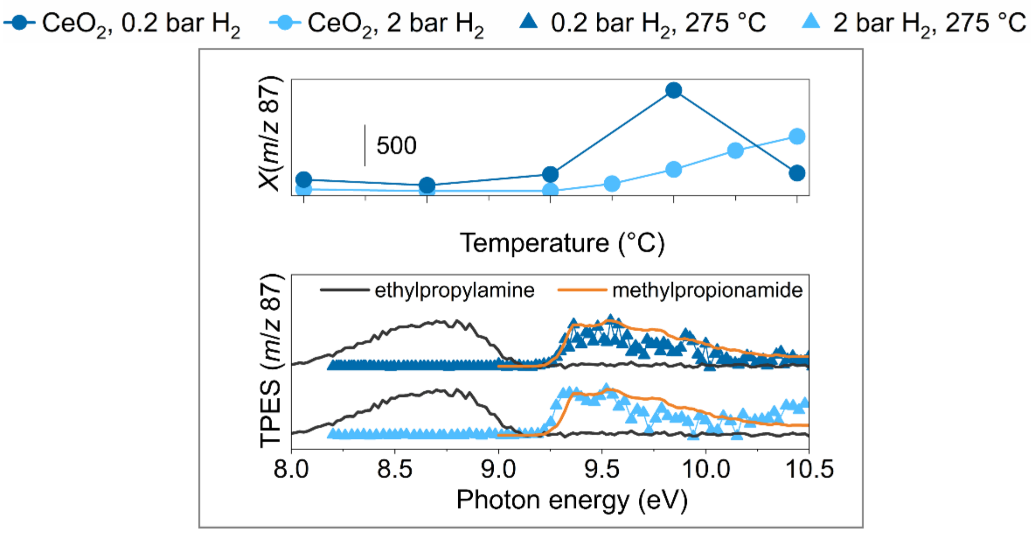

Figure S18. Temperature-dependent peak intensities and ms-TPES showing the formation of *m*/*z* 87 during the conversion of EPA catalyzed by CeO_2. The photoionization mass spectra were acquired at 9.5 eV. Reference spectra of ethylpropylamine and methylpropionamide were measured using pure samples in separate runs.

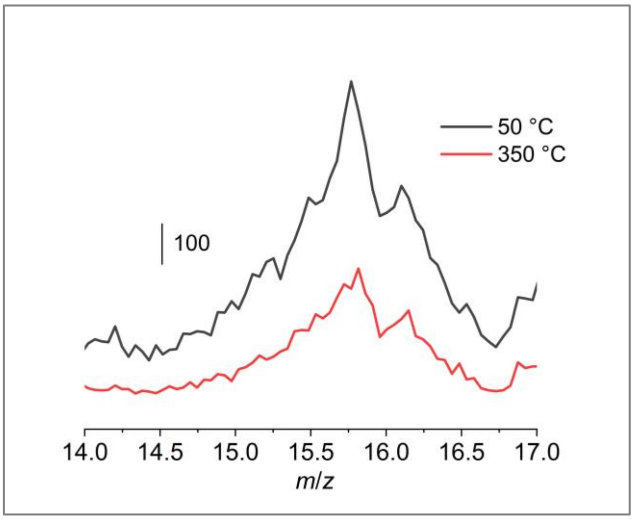

Figure S19. Photoionization mass spectra for the conversion of EPA catalyzed by CeO_2 under 0.2 bar H_2, acquired at 13 eV. The methane peak at *m*/*z* 16 is attributed to the residual background of the chamber.

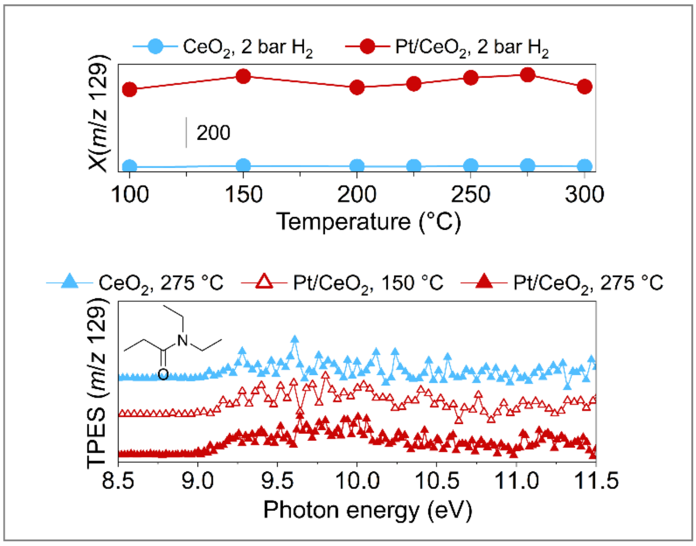

Figure S20. Temperature-dependent peak intensities and ms-TPES showing the formation of *m/z* 129 during the conversion of EPA catalyzed by Pt/CeO_2 and CeO_2 under 2 bar. The photoionization mass spectral intensities were acquired at 9.5 eV.

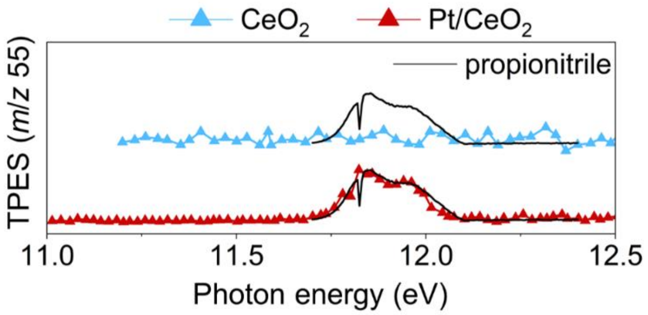

Figure S21. ms-TPES of *m*/*z* 55 for the conversion of propionamide with Pt/CeO_2 and CeO_2 at 200 °C under 2 bar H_2. Reference spectra of propionitrile were measured as pure samples in separate runs.

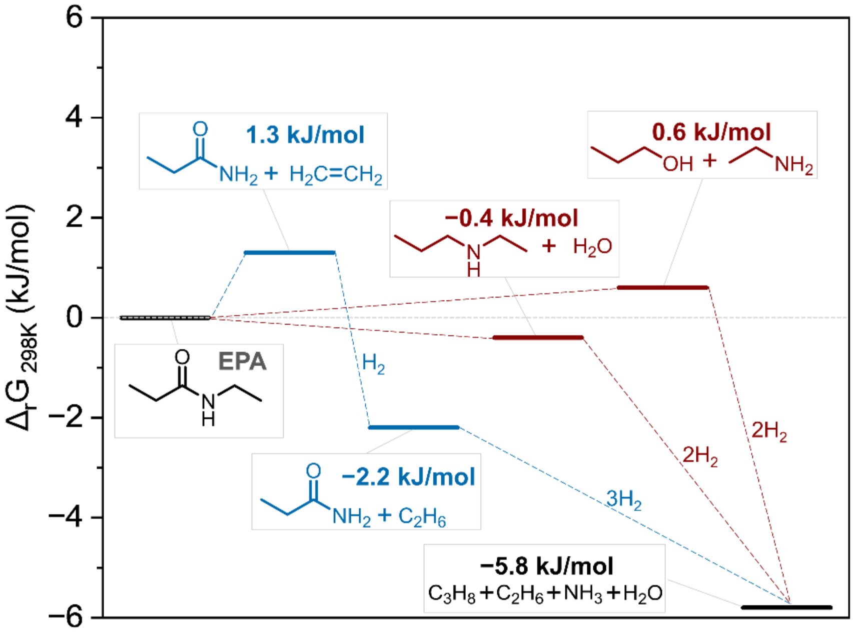

Figure S22. Favorability of different conversion pathways for EPA based on the computed reaction energies at 298 K. The formation energies for each species are provided in Table S1.

References

(1) Baer, T.; Tuckett, R. P. Advances in Threshold Photoelectron Spectroscopy (TPES) and Threshold Photoelectron Photoion Coincidence (TPEPICO). *Phys. Chem. Chem. Phys.* **2017**, *19* (15), 9698–9723.
(2) Montgomery, J. A., Jr.; Frisch, M. J.; Ochterski, J. W.; Petersson, G. A. A Complete Basis Set Model Chemistry. VI. Use of Density Functional Geometries and Frequencies. *The Journal of Chemical Physics* **1999**, *110* (6), 2822–2827.
(3) Montgomery, J. A., Jr.; Frisch, M. J.; Ochterski, J. W.; Petersson, G. A. A Complete Basis Set Model Chemistry. VII. Use of the Minimum Population Localization Method. *The Journal of Chemical Physics* **2000**, *112* (15), 6532–6542.
(4) Gaussian 16, Revision C.01, Frisch, M. J.; Trucks, G. W.; Schlegel, H. B.; Scuseria, G. E.; Robb, M. A.; Cheeseman, J. R.; Scalmani, G.; Barone, V.; Petersson, G. A.; Nakatsuji, H.; Li, X.; Caricato, M.; Marenich, A. V.; Bloino, J.; Janesko, B. G.; Gomperts, R.; Mennucci, B.; Hratchian, H. P.; Ortiz, J. V.; Izmaylov, A. F.; Sonnenberg, J. L.; Williams-Young, D.; Ding, F.; Lipparini, F.; Egidi, F.; Goings, J.; Peng, B.; Petrone, A.; Henderson, T.; Ranasinghe, D.; Zakrzewski, V. G.; Gao, J.; Rega, N.; Zheng, G.; Liang, W.; Hada, M.; Ehara, M.; Toyota, K.; Fukuda, R.; Hasegawa, J.; Ishida, M.; Nakajima, T.; Honda, Y.; Kitao, O.; Nakai, H.; Vreven, T.; Throssell, K.; Montgomery, J. A., Jr.; Peralta, J. E.; Ogliaro, F.; Bearpark, M. J.; Heyd, J. J.; Brothers, E. N.; Kudin, K. N.; Staroverov, V. N.; Keith, T. A.; Kobayashi, R.; Normand, J.; Raghavachari, K.; Rendell, A. P.; Burant, J. C.; Iyengar, S. S.; Tomasi, J.; Cossi, M.; Millam, J. M.; Klene, M.; Adamo, C.; Cammi, R.; Ochterski, J. W.; Martin, R. L.; Morokuma, K.; Farkas, O.; Foresman, J. B.; Fox, D. J. Gaussian, Inc., Wallingford CT, **2016**.
(5) Kimura, Katsumi. *Handbook of HeI Photoelectron Spectra of Fundamental Organic Molecules: Ionization Energies, Ab Initio Assignments, and Valence Electronic Structure for 200 Molecules*; Halsted Press (January 1, 1981).
(6) Kimura, K.; Katsumata, S.; Yamazaki, T.; Wakabayashi, H. UV Photoelectron Spectra and Sum Rule Consideration: Out-of-Plane Orbitals of Unsaturated Compounds with Planar-Skeleton Structure. *Journal of Electron Spectroscopy and Related Phenomena* **1975**, *6* (1), 41–52.
(7) Furuya, K.; Katsumata, S.; Kimura, K. Photoelectron Spectra of Acetone and Acetone Dimer. *Journal of Electron Spectroscopy and Related Phenomena* **1993**, *62* (3), 237–243.
(8) Locht, R.; Hottmann, K.; Hagenow, G.; Denzer, W.; Baumgartel, H. The Threshold-Photoelectron Spectrum of NH_3. *Chemical Physics Letters* **1992**, *190* (1), 124–129.
